# *In vivo* investigation of PEDV transmission via nasal infection: mechanisms of CD4^+^ T-cell-mediated intestinal infection

**DOI:** 10.1128/jvi.01761-24

**Published:** 2025-03-17

**Authors:** Qiu Zhong, Jiaxin Qi, Na Su, Zi Li, Chengcheng Wang, Hui Zeng, Ruiling Liu, Yuchen Li, Qian Yang

**Affiliations:** 1 MOE Joint International Research Laboratory of Animal Health and Food Safety, College of Veterinary Medicine, Nanjing Agricultural University 261674 , Nanjing, Jiangsu, China; 2 State Key Laboratory for Diagnosis and Treatment of Severe Zoonotic Infectious Diseases, Key Laboratory for Zoonosis Research of the Ministry of Education, Institute of Zoonosis, and College of Veterinary Medicine, Jilin University 623721 , Changchun, Jilin, China; Loyola University Chicago-Health Sciences Campus, Maywood, Illinois, USA

**Keywords:** porcine epidemic diarrhea virus, *in vivo* dissemination, CD4^+ ^T-cell, latent infection, integrin α4β7, intestinal homing, actin cytoskeleton rearrangement

## Abstract

The porcine epidemic diarrhea virus (PEDV), a highly pathogenic coronavirus, poses significant challenges to global swine agriculture with severe economic consequences. Our research reveals that in addition to known transmission routes, PEDV can be airborne, initially invading the nasal mucosa and subsequently being transported by dendritic cells and peripheral blood T cells, ultimately leading to intestinal disease in piglets. This study elucidates the cellular mechanisms behind the process, demonstrating how PEDV is internalized by CD4^+^ T cells after being transferred by dendritic cells, where it establishes a latent infection. Crucially, PEDV induces the upregulation of the integrin α4β7 homing receptor, facilitating the migration of these infected CD4^+^ T cells to the small intestine. Furthermore, our findings reveal that the activation of the α4β7-Rho-GTPases-Cofilin signaling pathway by PEDV reorganizes the actin cytoskeleton, enabling CD4^+^ T-cell transmigration through high endothelial venules into the intestinal mucosa, resulting in the infection of intestinal epithelial cells. These insights not only illuminate the molecular mechanisms PEDV employs to hijack CD4^+^ T cells for transmission from the respiratory tract to the intestine but also identify novel targets for therapeutic intervention, providing new perspectives for effectively preventing and managing PEDV infection with broader implications for controlling similar pathogens in diverse hosts.

**IMPORTANCE:**

Porcine epidemic diarrhea virus (PEDV), characterized by rapid transmission and widespread prevalence, poses a significant long-term threat to the global pig farming industry. Our previous research revealed that, in addition to the classic fecal-oral infection route, PEDV can invade through the nasal mucosa, leading to intestinal infection. This study further investigated the molecular mechanisms by which the virus is transported by T lymphocytes from the respiratory tract to the intestines. We found that PEDV establishes a latent infection in CD4^+^ T cells and promotes their intestinal homing by upregulating the homing receptor integrin α4β7. Additionally, we elucidated the activation of the integrin α4β7-mediated Rho-GTPase-Cofilin signaling axis by PEDV, which regulates pseudopod formation and facilitates CD4^+^ T-cell migration to the intestinal mucosal lamina propria post-homing. This study elucidates the mechanism underlying the lymphocyte-dependent dissemination of PEDV following nasal infection, providing new insights into strategies for preventing PEDV invasion.

## INTRODUCTION

In recent years, the recurrent emergence of coronaviruses has posed a significant threat to both human and animal health (1, 2). Among these viruses, the porcine epidemic diarrhea virus (PEDV) is one of the most detrimental enteric coronaviruses affecting swine, leading to substantial economic losses in the pig farming industry worldwide (3, 4). Despite considerable advancements in understanding the pathogenesis of PEDV, as well as the development of detection techniques and vaccines, the disease remains endemic in numerous regions globally.

PEDV can be transmitted through aerosols, exhibiting a remarkably rapid spread once an outbreak occurs. In previous studies, we have preliminarily elucidated the mechanism by which PEDV causes intestinal infection in piglets via airborne transmission (5–7). Our findings indicate that the virus exploits submucosal DCs (dendritic cells, DCs) invading the nasal mucosa and is subsequently transferred to peripheral blood T cells via virological synapses. Subsequently, the virus-loaded T cells reach the intestine through blood circulation, leading to intestinal infection via cell-cell contact. However, the key molecules and specific signaling pathways involved in this process remain undefined, presenting significant challenges in blocking the nasal infection route of PEDV.

The migration of immune cells within the body is precisely regulated by the selective expression of specific homing receptors on their surfaces (8, 9). This crucial process is mediated by interactions between these homing receptors and adhesion molecules present in the vascular endothelial cells of targeted tissues and organs, enabling lymphocytes to navigate to their designated locations. CD4^+^ T cells use various homing receptors for localization and migration. Specifically, L-selectin enables naïve T cells to enter lymph nodes from the bloodstream, ensuring effective settlement in secondary lymphoid organs. Furthermore, the CCR7 and CXCR4 chemokine receptors play distinct roles: CCR7 directs T cells back to lymph nodes from peripheral tissues, while CXCR4 guides them to inflammatory sites, ensuring efficient immune surveillance and response. In addition, integrins play a vital role; integrin αLβ2 aids T-cell binding to antigen-presenting cells via ICAM-1, which is crucial for T-cell activation and the formation of immunological synapses (10). Moreover, integrins α4β7 and αEβ7, through binding to mucosal address cell adhesion molecule-1 (MAdCAM-1), facilitate T-cell entry into gut-associated lymphoid tissue and epithelial tissues, thus playing key roles in mucosal immunity (10, 11).

Previous studies have shown that some viruses enhance infection by modulating immune cell migration via integrin expression. HCMV (human cytomegalovirus, HCMV) increases dendritic cell (mDC) adhesion and reduces their migration toward CCL19 by regulating β2 integrin (lymphocyte function-associated antigen, LFA-1), hindering their movement to secondary lymphoid organs and evading immune surveillance (12). HCMV, on the other hand, activates the integrins LFA-1 and VLA-4 (very late antigen-4, VLA-4), enhancing immune cell adhesion to endothelial cells and promoting viral migration into the bloodstream, thereby facilitating viral dissemination and expansion within the host (13). Therefore, whether PEDV also influences CD4^+^ T-cell migration by regulating integrin activity warrants further investigation.

The migration of lymphocytes hinges on the formation of invasive pseudopodia, involving the generation of actin-rich pseudopodia, lamellipodia extension, and contractile uropod formation (14, 15). Central to this process is the reorganization of the actin cytoskeleton, which is composed of filaments formed by G-actin monomer polymerization. These monomers bind ATP and assemble into polarized, double-helical F-actin filaments, creating “barbed” and “pointed” ends. Under steady-state conditions, a dynamic equilibrium of monomer binding and dissociation at both ends maintains the stability of the filament length. During the reorganization, the barbed end elongates with continuous actin subunit addition, while the pointed end shortens due to subunit dissociation, enabling dynamic remodeling. Actin-binding proteins, such as cofilin, play a critical role in this process. Cofilin binds and severs actin filaments, promoting depolymerization, whereas phosphorylated cofilin activity is inhibited, facilitating filament assembly (16, 17). The Arp2/3 (actin-related protein 2/3 complex, Arp2/3) complex promotes actin polymerization by inducing short filament branching into dendritic structures (18) while Formin binds to the barbed end, elongating linear structures and preventing depolymerization (19).

In summary, our study aims to establish both an *in vivo* piglet model and an *in vitro* T lymphocyte culture model to systematically elucidate the molecular mechanisms by which PEDV regulates homing molecules on the surface of peripheral blood T cells and the cell microfilament cytoskeleton following invasion. By targeting these regulatory pathways, we aim to understand how PEDV directs these cells to migrate to the intestines, leading to intestinal infection. The findings provide potential targets for preventing the internal transmission of PEDV from the nasal cavity (a nonsusceptible site) to the intestines (a susceptible site) and offer significant insights into the transmission mechanisms of other viruses with similar characteristics.

## RESULTS

### PEDV establishes latent infection in peripheral blood CD4^+^ T cells following nasal inoculation

**Fig 1:**
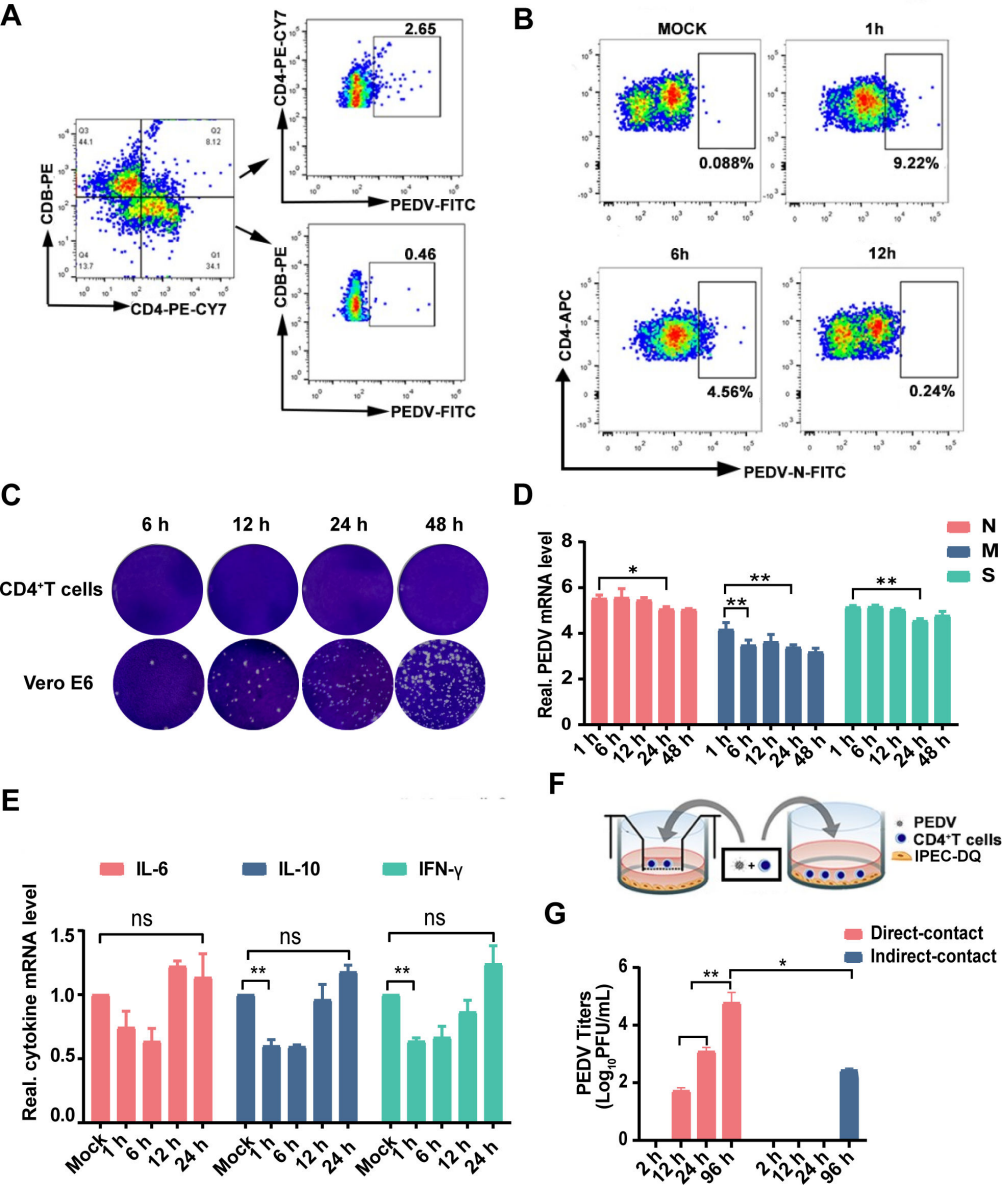
Nasal mucosal invasion by PEDV primarily infects CD4^+^ T cells and establishes a latent infection. (**A**) For Fluorescent-Activated Cell Sorting (FACS) analyses, individual cells isolated from the pig’s blood sample co-culture with PEDV-infected DC. Then, individual cells were gated based on CD4^+^ and CD8^+^, and viral infection was detected by PEDV N protein staining, *n* =  6 from 3 piglets per group. (**B**) For FACS analyses, individual cells isolated from the pig’s blood sample co-culture with PEDV-infected DC at the indicated time. Flow cytometry was used to detect viral proteins in CD4^+^ T cells. (**C**) Following infection of CD4^+^ T cells with PEDV, a plaque assay was performed to detect the release of the virus in the supernatant of CD4^+^ T cells. (**D**) Quantitative real-time PCR (qRT-PCR) was used to detect viral genomes in CD4^+^ T cells. (**E**) qRT-PCR was used to measure the interferons in CD4^+^ T cells post-PEDV infection. (**F**) Schematic of the model to study PEDV-carrying CD4^+^ T cells transmission of the virus to IPEC-DQ. CD4^+^ T cells were infected with PEDV for 2 h, then, the remaining CD4^+^ T cells were cocultured with IPEC-DQ by two methods (contact and noncontact coculture). (**G**) The viral titers in the supernatant were measured at indicated times post-co-culture. All the data are presented as the means ± SDs, and comparisons were performed using one-way analysis of variance (ANOVA). *P < 0.05, **P < 0.01; ns, not significant. The results are from at least three different experiments.

Our previous studies have shown that intranasal administration of PEDV in piglets induces typical diarrhea and transient infection of the nasal epithelium (5). Further research revealed that PEDV exploits the transepithelial capture capability of DCs to enter the nasal mucosa and subsequently transfer the virus to peripheral blood T cells. In this study, flow cytometry analysis demonstrated that following intranasal inoculation with PEDV, the virus predominantly transferred to CD4^+^ T cells (Fig. 1A). To further confirm these *in vivo* findings, we utilized magnetic bead separation to isolate CD4^+^ T cells from the peripheral blood of piglets, and then inoculated them with PEDV at a multiplicity of infection, observing growth characteristics consistent with latent viral infection as described by Delwart in “Principles of Virology” (20). Specifically, the percentage of PEDV entering CD4^+^ T cells reached a peak at 1 hour post-infection (1 hpi) and then gradually decreased (Fig. 1B). In addition, no progeny virus was detected in CD4T^+^ after PEDV infection (Fig. 1C). Viral M (membrane, M), N (nucleocapsid, N), and S (spike, S) structural genes remained stable in quantity (Fig. 1D), while genes associated with extensive viral replication and assembly showed no transcription or were present at very low levels (Ct values of nsp1, nsp5, PLP2, and ORF3 all >30) (Table S3). Additionally, PEDV effectively suppressed interferon production in CD4^+^ T cells (Fig. 1E). Moreover, CD4^+^ T cells harboring viral genetic components were capable of transmitting the virus to porcine intestinal epithelial cells (IPEC-DQ), leading to PEDV infection in these cells (Fig. 1F and G).

### PEDV enhances the expression of the gut-homing receptor integrin α4β7 on CD4^+^ T cells

**Fig 2:**
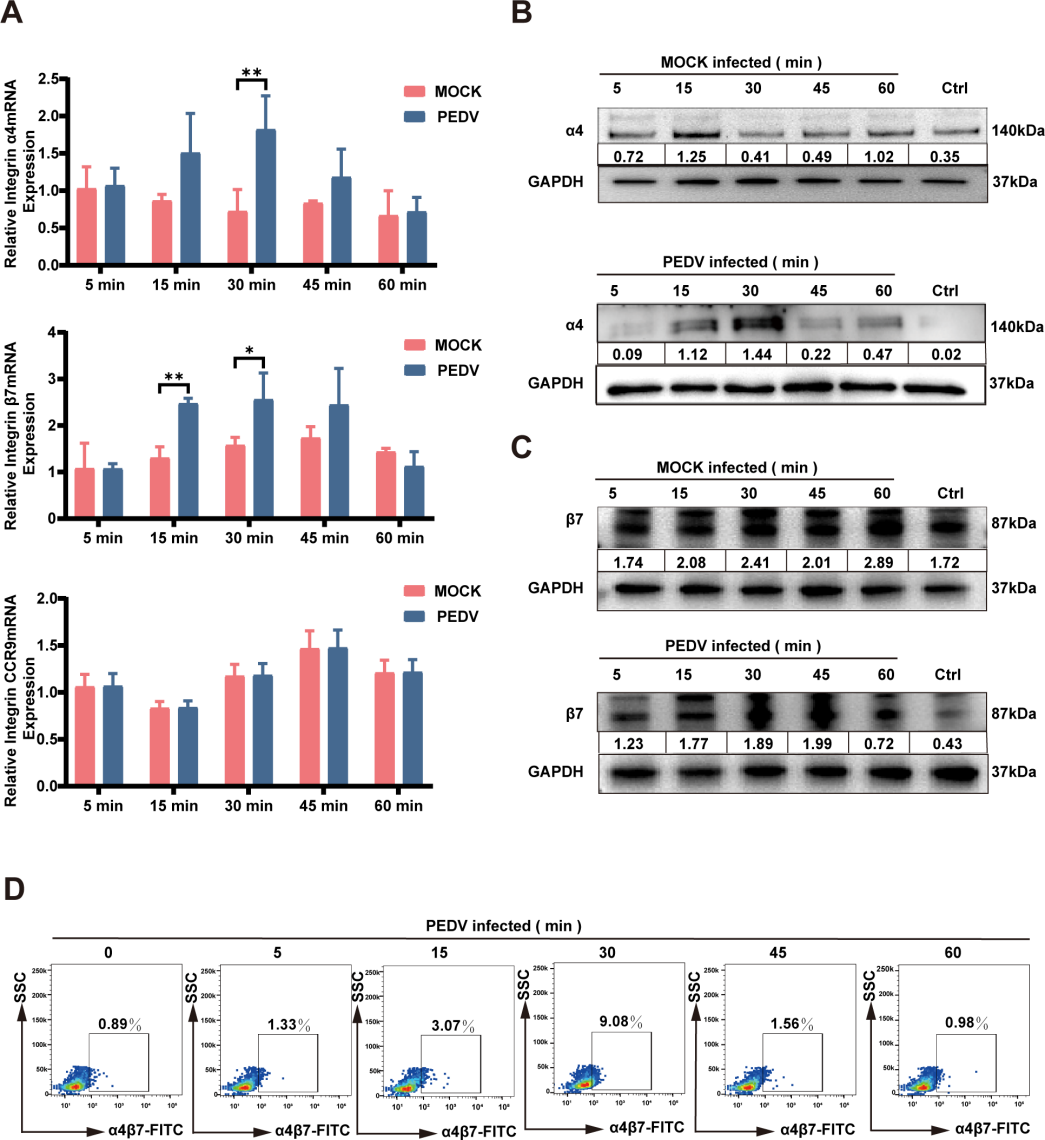
Invasion of CD4^+^ T cells by PEDV activates integrin α4β7. (**A–D**) CD4^+^ T cells were inoculated with PEDV (MOI of 1), then cells were collected at indicated times. The expression of α4β7 was detected by qRT-PCR (**A**), western blotting (**B and C**), and flow cytometry (**D**). All the data are presented as the means ± SDs, and comparisons were performed using one-way ANOVA. *P < 0.05, **P < 0.01. The results are from at least three different experiments.

The migration of T cells within the body is meticulously regulated by the selective expression of specific homing receptors on their surfaces (8). Integrin α4β7 and the chemokine receptor CCR9 play pivotal roles in directing CD4^+^ T cells to the small intestine (7, 21). Specifically, integrin α4β7 binds to MAdCAM-1 on intestinal endothelial cells, thereby facilitating CD4^+^ T-cell entry into intestinal lymphoid tissues, while CCR9 interacts with its ligand CCL25, which is abundantly expressed in the intestinal epithelium (10, 11). In this study, we investigated the expression of integrin α4β7 and CCR9 in CD4^+^ T cells following infection with PEDV. In subsequent experiments, CD4^+^ T cells were infected with PEDV at a multiplicity of infection (MOI) of 1.0 PFU/cell and harvested at the designated times post-infection. The results of quantitative real-time PCR (qRT-PCR) indicated that the transcription of integrin α4β7 in CD4^+^ T cells was upregulated from 15 to 45 min post-infection, and reached a peak at 30 min, while the transcription of CCR9 remained unaffected (Fig. 2A). The upregulation of integrin α4β7 transcription was further substantiated by western blotting (Fig. 2B and C) and flow cytometry (Fig. 2D) analyses. These analyses revealed a significant increase in α4β7 integrin protein expression as early as 15 min post-infection, with expression peaking at 30 min post-infection. These findings suggest that PEDV infection induces a marked increase in integrin α4β7 expression in CD4^+^ T cells, highlighting a potential mechanism for the targeted migration of these cells to intestinal tissue during infection.

### Integrin α4β7 played a key role in the intestinal migration of CD4^+^ T cells induced by PEDV

**Fig 3:**
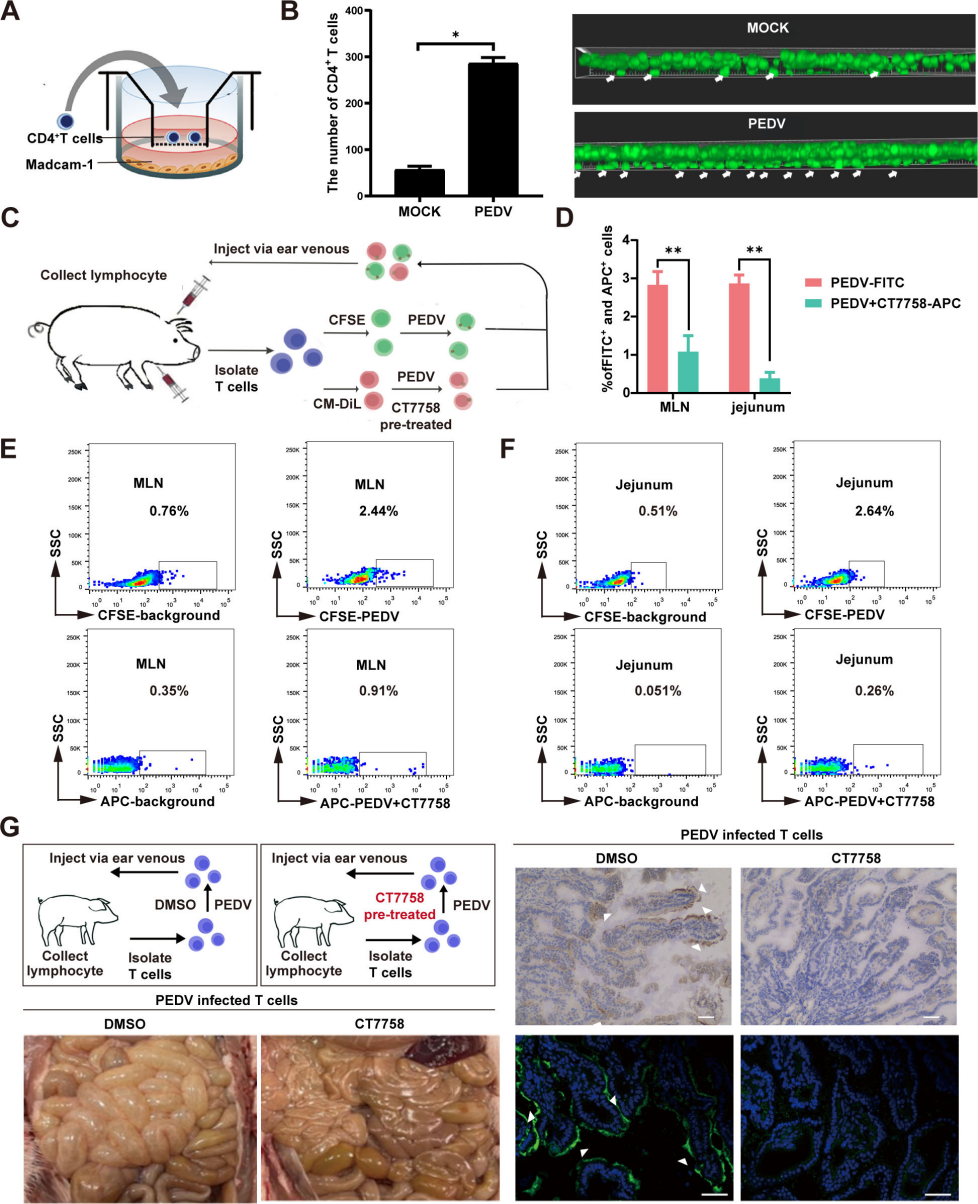
The role of integrin α4β7 in CD4^+^ T cells targeting and migration to the intestinal tract. (**A**) Diagram of the Transwell migration assay . A Transwell chamber is placed in a 24-well cell plate, with 100 µL of 50% Matrigel added to the upper chamber. Infected PEDV CD4^+^ T cells (10^6^ /mL) are added to the upper chamber, and prokaryotic-expressed chemokines MAdCAM-1 and CCL25 are added to the lower chamber. (**B**) The migration of CD4^+^ T cells was visualized using CFSE staining 12 h after PEDV infection. The filters were processed and viewed using confocal laser scanning microscopy (CLSM). The white arrowheads represent migrated CD4^+^ T cells. The bar chart is a statistical quantification of the number of migrated CD4^+^ T cells. (**C**) Schematic of the competitive reinfusion experiment with piglets using PEDV-infected T cells treated with or not the inhibitor CT7758. The T cells of the PEDV-infected group labeled CFSE. The T cells of the inhibitor pretreated group labeled CM-DiD. (**D**) The quantified FACS results are shown. (**E and F**) After a competitive reinfusion experiment, flow cytometry is used to detect the number of CFSE and CM-DiD labeled T cells in MLN (**E**) and jejunum (**F**). (**G**) Piglets are divided into two groups for autologous reinfusion of either PEDV-infected T cells or those treated with the inhibitor. Gross lesions of the intestine in piglets 24 h after autologous reinfusion; immunological fluorescence assay (scale = 2 µm) and immunohistochemistry (scale = 50 µm) results showing the distribution pattern of PEDV in the jejunum. PEDV-positive cells are indicated by white arrows. All the data are presented as the means ± SDs, and comparisons were performed using one-way ANOVA. *P < 0.05, **P < 0.01. The results are from at least three different experiments.

To investigate the impact of integrin α4β7 on the regulation of CD4^+^ T-cell migration by PEDV, cell migration assays were conducted using a Matrigel-coated Transwell chamber (Fig. 3A). Confocal microscopy revealed a substantial increase in the migration of green fluorescent-labeled CD4^+^ T cells to the lower chamber in the PEDV-infected group compared to the mock group, indicating that PEDV infection significantly enhances CD4^+^ T-cell migration (Fig. 3B). Additionally, a competitive T cells autotransfusion assay was performed to examine the role of integrin α4β7 in facilitating the targeted intestinal migration of T cells by PEDV. The T cells were isolated from the peripheral blood lymphocytes of piglets and subsequently treated with the integrin inhibitor CT7758 (inhibitor group) for 1 h prior to PEDV inoculation, with DMSO used as the solvent control (mock group). The T cells from the mock and inhibitor-pretreated groups were labeled with the fluorescent dyes CFSE (green) and CM-DiD (red), respectively. These labeled cells were then mixed and adoptively transferred into piglets (autologous) via injection into the anterior vena cava (Fig. 3C). After 24 h, the distribution of labeled-T cells in the mesenteric lymph nodes (MLN) and mesenteric jejunal mucosa was detected by flow cytometry. Consistent with our previous reports, flow cytometry revealed that a significant number of CFSE-labeled T cells migrated to the mesenteric jejunal mucosa 24 h after infusion, indicating that PEDV can promote the migration of T cells to the intestine. However, pretreatment with the inhibitor CT7758 significantly reduced the number of T cells (CM-DiD-labeled) in these areas (Fig. 3D through F).

Additionally, when T cells from the mock group and the inhibitor-pretreated group were reinfused into separate piglets, the mock group exhibited significant diarrhea symptoms. Postmortem examination revealed thinning of the small intestinal wall and substantial fluid accumulation within the intestine. In contrast, piglets in the inhibitor-pretreated group displayed no overt clinical symptoms, and their small intestines appeared normal. Immunofluorescence and immunohistochemistry analyses showed that in the PEDV-infected group, the small intestinal villi were severely damaged, with necrosis and detachment of intestinal epithelial cells, along with a high presence of viral antigens in the jejunum. Conversely, piglets treated with the inhibitor maintained intact intestinal epithelial structures with no detectable viral antigens (Fig. 3G).

### PEDV activates actin cytoskeleton regulation pathways in CD4^+^ T cells via integrin α4β7

Transcriptome sequencing analysis was conducted to elucidate the major molecular mechanisms by which PEDV regulates CD4^+^ T cells through the modulation of integrin α4β7. As shown in Fig. S1A, 424 differentially expressed genes (DEGs) were identified in the PEDV-infected group compared with the mock-infected group, with 280 downregulated genes and 145 upregulated genes. However, in comparison to the PEDV-infected group, pretreatment with CT7758 (an integrin α4β7 inhibitor) led to the upregulation of 558 genes and the downregulation of 392 genes (Fig. S1B). The heatmap displays DEGs across the various treatment groups, with clustering analysis revealing that CT7758 treatment partially restored the transcriptomic profile of PEDV-infected CD4^+^ T cells to resemble that of uninfected cells (Fig. S1C). Venn diagram analysis revealed 551 DEGs that were exclusively expressed in the PEDV-infected group compared to the mock-infected group. Conversely, 551 DEGs were exclusively expressed in the inhibitor-treated group compared to the PEDV-infected group. In these two comparisons, only 6 and 12 overlapping DEGs were identified, respectively (Fig. S1D and E). Gene Ontology and Kyoto Encyclopedia of Genes and Genomes enrichment analyses revealed that, compared to those in the mock-infected group, the genes upregulated in PEDV-infected CD4^+^ T cells were enriched in the TGF-β, extracellular matrix, T-cell receptor, and IgA production signaling pathways (Fig. S1F and G). Conversely, compared to the PEDV-infected group, the inhibitor-pretreated group displayed significant downregulation of the immune response, inflammatory response, chemokine, TOLL-like receptor, and NF-κB signaling pathways (Fig. S1H and I).

**Fig 4:**
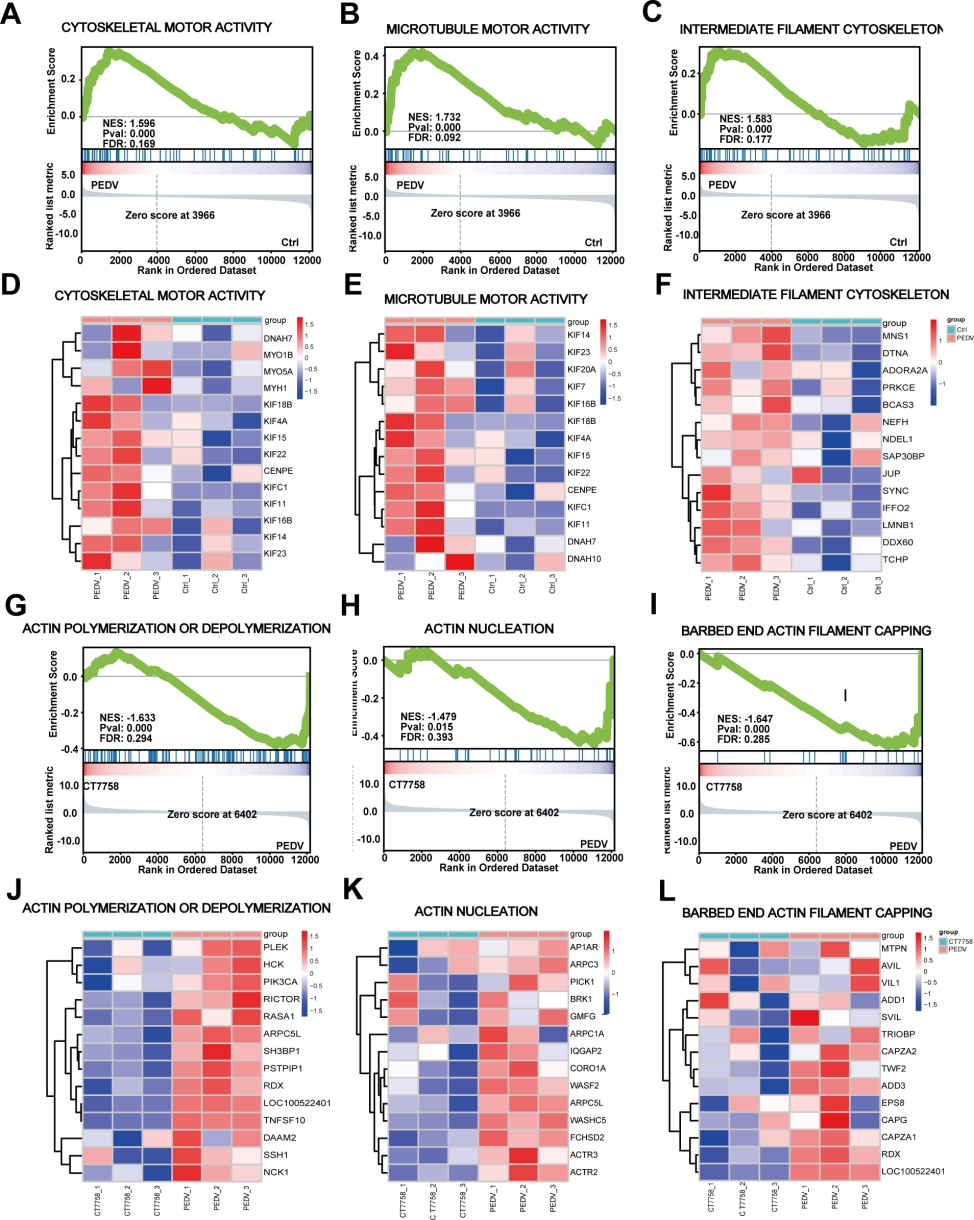
PEDV regulates multiple signaling pathways related to the CD4^+^ T cells microfilament cytoskeleton via integrin α4β7. (**A–C**) GSEA of DEGs associated with the cytoskeleton (**A**), microtubules (**B**), and intermediate filament cytoskeleton (**C**) between the mock-infected and PEDV-infected groups. (**D–F**) Heatmaps of gene expression clustering for pathways related to the cytoskeleton (**D**), microtubules (**E**), and intermediate filament cytoskeleton (**F**) between the mock-infected and PEDV-infected groups. (**H and I**) GSEA of DEGs associated with actin polymerization or depolymerization (**G**), actin filament capping (**H**), and actin nucleation (**I**) between the PEDV and inhibitor-pretreated groups. (**J–L**) Heatmaps of gene expression clustering for pathways related to actin polymerization or depolymerization (**J**), actin filament capping (**K**), and actin nucleation (**L**) between the PEDV-infected and inhibitor-pretreated groups.

The migration of immune cells is primarily governed by dynamic changes in the actin cytoskeleton. Further gene set enrichment analysis (GSEA) indicated that PEDV infection significantly activated signaling pathways regulating actin filaments (Fig. 4A), microtubules (Fig. 4B), and intermediate filaments (Fig. 4C), all of which collectively constitute the cellular cytoskeleton. Further heatmap analysis revealed significant upregulation of the transcription levels of key genes involved in these pathways, including members of the KIF family (KIF11, KIF14, KIF23, KIF16B), MYO family (MYO5A, MYO1B), and DNAH family (DNAH7, DNAH10), all of which play critical roles in cytoskeletal regulation (Fig. 4D through F).

The dynamic changes in the actin cytoskeleton involve polymerization and depolymerization, which can be divided into two stages: a slow nucleation phase and a rapid elongation phase. During the nucleation phase, oligomers composed of at least 2–3 actin monomers are formed, catalyzed by nucleating proteins such as the Arp2/3 complex. In the elongation phase, actin monomers rapidly assemble using the energy released from ATP hydrolysis, forming a cap of actin-ATP subunits that stabilizes the filaments and facilitates further assembly.

Our study revealed that treatment with CT7758, an integrin α4β7 inhibitor, significantly inhibited the signaling pathways related to actin polymerization and depolymerization (Fig. 4G), actin filament capping (Fig. 4H), and actin nucleation (Fig. 4I) induced by PEDV infection. Notably, the expression of genes associated with the cytoskeleton, including those in the ARP family (ARPC5L, ARPC1A, and ARPC3), WASF family (WASF2), and actin-binding proteins (ADD1 and ADD3), were significantly downregulated (Fig. 4J through L). These findings suggest that PEDV may induce cytoskeletal rearrangement through integrin α4β7, thereby modulating the intestinal migration of CD4^+^ T cells.

### PEDV-induced actin cytoskeleton rearrangement enhances the migration of CD4^+^ T cells by promoting filopodium formation

**Fig 5:**
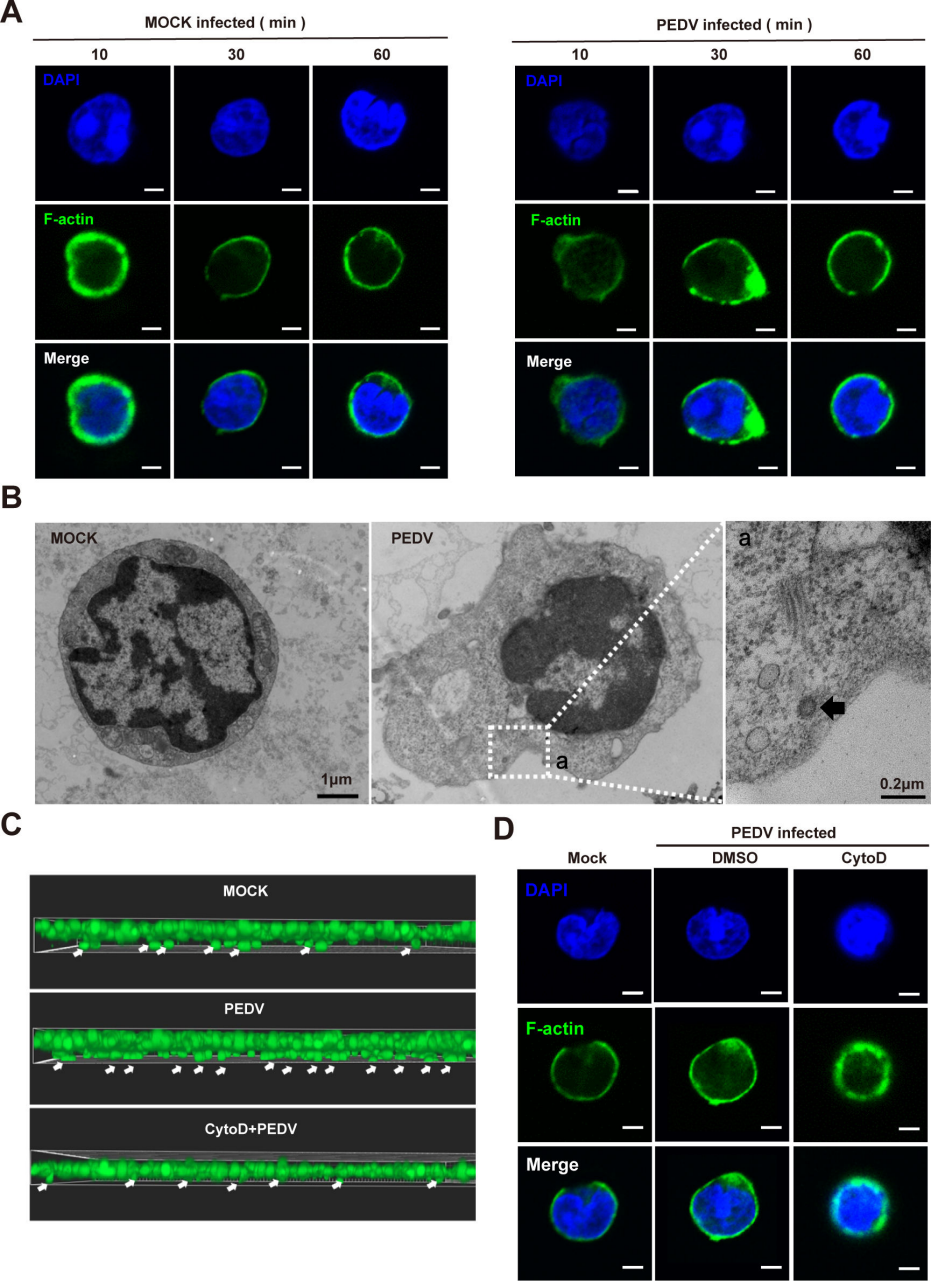
PEDV promotes cellular migration by inducing rearrangement of CD4^+^ T cells microfilament cytoskeleton. (**A and B**) CD4^+^ T cells were inoculated with PEDV (MOI of 1), then cells were collected at indicated times. (**A**) Alterations of the cytoskeleton were stained with phalloidine (green) and cell nuclei were stained with DAPI (blue). The cytoskeleton is viewed via CLSM (scale = 2 µm). (**B**) Alterations in the cellular microfilament cytoskeleton are also examined using TEM, and PEDV particles within CD4^+^ T cells are observed (black arrowheads). (**C**) CD4^+^ T cells are treated with the actin-depolymerizing agent CytoD for 1 h, placed in a Transwell chamber, and then infected with PEDV (MOI of 1) for 30 min. Images of migrating CD4^+^ T cells are captured via CLSM. (**D**) Alterations of the cytoskeleton were stained with phalloidine (green), and cell nuclei were stained with DAPI (blue). The cytoskeleton is viewed via CLSM (scale = 2 µm). All results are representative of three independent experiments.

Transcriptome sequencing results indicated that PEDV may induce actin cytoskeleton rearrangement via integrin α4β7, thereby regulating CD4^+^ T-cell migration. The core component of the actin cytoskeleton, F-actin, plays a crucial role in the formation of lamellipodia. These dynamic structures enable immune cells to respond to chemical gradients and other signals, adjusting their migration direction and speed for effective positioning and immune response (10, 11). To test this hypothesis, we employed confocal microscopy to observe the impact of PEDV infection on the actin cytoskeleton of CD4^+^ T cells. After 30 min post-infection, F-actin (green) significantly aggregated and formed lamellipodia, and these structures gradually returned to their original state after 1 h (Fig. 5A). Transmission electron microscopy (TEM) further corroborated these findings, revealing notable morphological changes and prominent lamellipodia formation in PEDV-infected CD4^+^ T cells compared to those in the mock-infected group (Fig. 5B). Additionally, to confirm the role of PEDV in modulating CD4^+^ T-cell migration via actin cytoskeleton rearrangement, we pretreated CD4^+^ T cells with the actin-depolymerizing agent cytochalasin D (CytoD). CytoD effectively inhibited PEDV-induced lamellipodia formation and significantly suppressed the enhanced migration of CD4^+^ T cells caused by PEDV infection (Fig. 5C and D).

### PEDV-induced rearrangements of the actin cytoskeleton in CD4^+^ T cells via integrin α4β7/LIMK/Cofilin signaling

**Fig 6:**
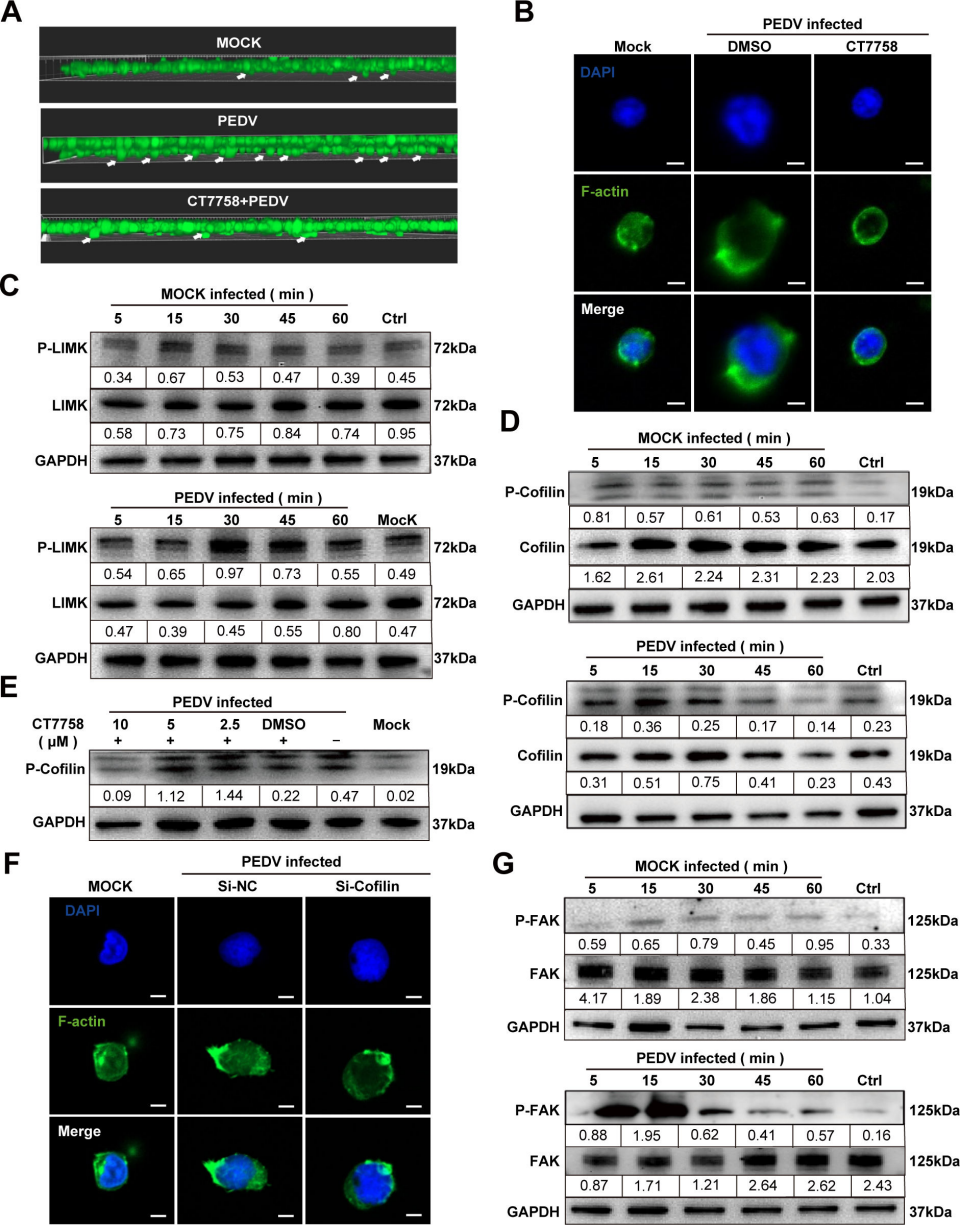
PEDV induces rearrangement of the microfilament cytoskeleton in CD4^+^ T cells through the integrin α4β7/LIMK/Cofilin signaling pathway. (**A and B**) CD4^+^ T cells were treated with the α4β7-specific inhibitor for 1 h, then inoculated with PEDV (MOI of 1) and CD4^+^ T cells for 30 min. (**A**) The CD4^+^ T cells were placed in the upper chamber of the Transwell chamber, and the migration of CD4^+^ T cells was observed by CFSE staining 12 h after PEDV infection. The filters were processed and viewed via CLSM. The white arrowheads represent migrated CD4^+^ T cells. (**B**) Alterations of the cytoskeleton were stained with phalloidine (green), and cell nuclei were stained with DAPI (blue). The cytoskeleton is viewed via CLSM (scale = 2 µm). (**C and D**) CD4^+^ T cells were inoculated with PEDV (MOI of 1), then lysates of PEDV-infected cells were collected at indicated times. Levels of total and phosphorylated LIMK (**C**) and cofilin (**D**) also were detected by western blotting. (**E**) CD4^+^ T cells pre-pretreated with the α4β7 inhibitor were tested for levels of phosphorylated cofilin after infection with PEDV (MOI of 1) by western blotting. (**F**) Cells were transfected with short interfering RNA (siRNA) targeting cofilin. At 24 h post-transfection and 1 h post-PEDV infection, alterations of the cytoskeleton were stained with phalloidine (green), and cell nuclei were stained with DAPI (blue). The cytoskeleton is viewed via CLSM (scale = 2 µm). (**G**) Activation of focal adhesion kinase (FAK) during PEDV infection. CD4^+^ T cells were inoculated with PEDV (MOI of 1), then lysates of PEDV-infected cells were collected at indicated times. The expression of the total and phosphorylated protein of FAK also was detected by western blotting. All results are representative of three independent experiments.

Our further study aimed to determine whether PEDV-induced actin cytoskeleton rearrangement in CD4^+^ T cells depends on integrin α4β7. Confocal microscopy revealed that pretreatment with the integrin α4β7 inhibitor CT7758 effectively blocked PEDV-induced actin cytoskeleton rearrangement and pseudopodia formation. Additionally, this pretreatment significantly inhibited the PEDV-enhanced migration of CD4^+^ T cells, indicating that integrin α4β7 plays a crucial role in these processes (Fig. 6A and B).

Cofilin, a key actin-binding protein, promotes F-actin depolymerization, while LIMK, a protein kinase, regulates cofilin activity through phosphorylation. Specifically, cofilin facilitates actin depolymerization and repolymerization, whereas LIMK phosphorylates cofilin, inhibiting its depolymerization activity. Together, these proteins complementarily regulate actin dynamics, thus influencing cell shape and movement. Our study revealed that PEDV infection caused a significant increase in cofilin phosphorylation (inactivation) in CD4^+^ T cells within 30 min, followed by a gradual decrease in phosphorylation (reactivation) (Fig. 6C and D). This temporal change in cofilin activity closely correlates with the dynamic changes observed in actin filaments. Further data demonstrated that the integrin α4β7 inhibitor CT7758 inhibited the PEDV-induced regulation of cofilin activity in CD4^+^ T cells in a concentration-dependent manner (Fig. 6E). Furthermore, cofilin-specific short interfering RNA (siRNA) effectively blocked PEDV-induced rearrangement of the actin cytoskeleton and pseudopodia formation in CD4^+^ T cells (Fig. S3), suggesting that PEDV primarily regulates the actin cytoskeleton through modulation of cofilin activity (Fig. 6F). Additionally, our research revealed that PEDV infection leads to dynamic changes in LIMK phosphorylation in CD4^+^ T cells, characterized by brief activation followed by rapid inactivation. This pattern suggested that PEDV may regulate cofilin activity through LIMK.

Moreover, we investigated the impact of PEDV infection on focal adhesion kinase (FAK), a molecule regulated by integrin interactions. Our findings indicated that FAK activation in CD4^+^ T cells occurred within 30 min post-infection, paralleling the observed changes in the actin cytoskeleton (Fig. 6G). This finding suggested that integrin α4β7 may regulate the activities of cofilin and LIMK via FAK, highlighting a potential pathway through which PEDV influences cytoskeletal dynamics and CD4^+^ T cell migration.

### Involvement of the Rho family of GTPases in PEDV-induced LIMK/Cofilin signaling in CD4^+^ T cells

**Fig 7:**
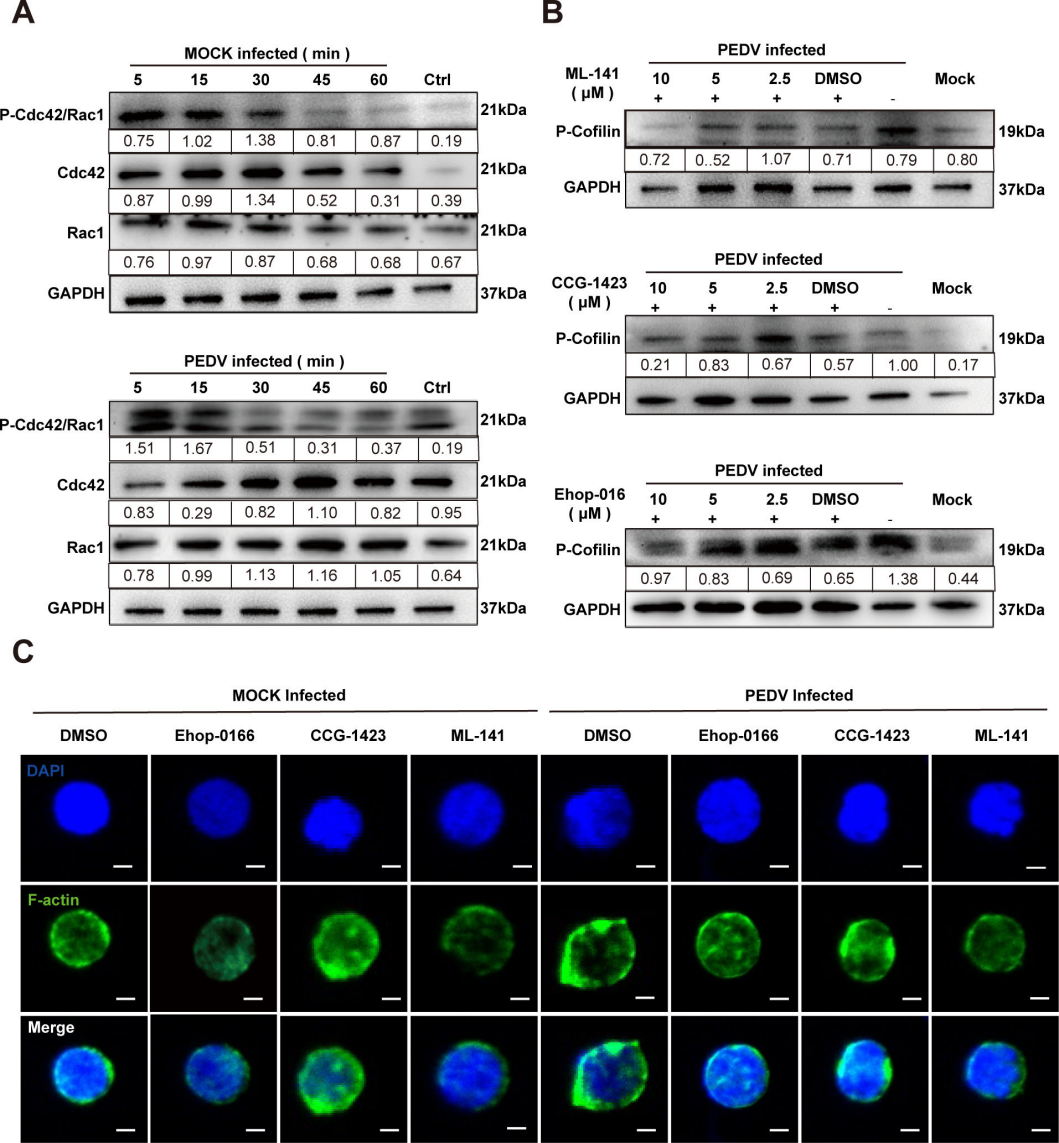
The Rho-GTPase family is involved in the regulation of P-cofilin following PEDV invasion. (**A**) Activation of Cdc42 and Rac1 during PEDV infection. CD4^+^ T cells were inoculated with PEDV (MOI of 1), then lysates of PEDV-infected cells were collected at indicated times. The expression of total and phosphorylated proteins of Cdc42 and Rac1 were detected by western blotting. The expression level of the phosphorylated protein of cofilin was detected by western blotting. (**B and C**) CD4^+^ T cells were treated with different concentrations of inhibitors and then infected with PEDV. (**B**) The expression of the phosphorylated protein of cofilin was detected by western blotting, and (**C**) alterations in the cellular microfilament cytoskeleton (green) were evaluated via CLSM. Cell nuclei were stained with DAPI (blue) (scale = 2 µm).

The activity of cofilin and LIMK is finely regulated by various signaling pathways, particularly those mediated by the Rho family of GTPases. The Rho family of GTPases, represented by members such as RhoA, Rac1, and Cdc42, act as molecular switches that cycle between inactive GDP-bound and active GTP-bound states, regulating the assembly and disassembly of the cellular actin cytoskeleton and influencing cell morphology, movement, and division. Therefore, this study aimed to investigate the involvement of Rho family GTPases in the regulation of cofilin activity induced by PEDV. Western blotting analysis revealed that the activities of Cdc42-GTPase and Rac1-GTPase were transiently induced by PEDV during the initial phases of infection at 30 min (Fig. 7A). However, during this period, the activity of RhoA in CD4^+^ T cells was not significantly altered.

Further data demonstrated that the Rac1 GTPase inhibitor EHop-016 and the Cdc42 GTPase inhibitor ML-141 inhibited the PEDV-induced regulation of cofilin activity in CD4^+^ T cells in a concentration-dependent manner (Fig. 7B). In contrast, pretreatment with the RhoA GTPase inhibitor CCG-1423 did not affect the virus-induced regulation of cofilin activity. The confocal microscopy results further confirmed these findings. As illustrated in Fig. 7C, inhibitors of the Rac1 and Cdc42 GTPases reversed the alterations in the microfilament cytoskeleton induced by PEDV infection, while the RhoA GTPase inhibitor did not. These results indicate that the Rac1 and Cdc42 GTPases play critical roles in the PEDV-induced regulation of cofilin activity in CD4^+^ T cells.

### PAK1/ROCK, which acts as downstream effectors of Cdc42/Rac1, plays a role in the activation of the cofilin signaling pathway subsequent to PEDV invasion

Activated Cdc42 and Rac1 further activate the downstream serine/threonine kinases PAK1 and ROCK, promoting cytoskeletal remodeling, cell migration, polarity formation, and other essential biological processes related to cell morphology and movement (22). PAK1 regulates actin dynamics by phosphorylating LIM kinase and actin-binding proteins, while ROCK facilitates cell contraction, adhesion, and directional migration by regulating actin-myosin interactions, ensuring precise control of the cytoskeleton and accurate execution of cellular behaviors (22).

**Fig 8:**
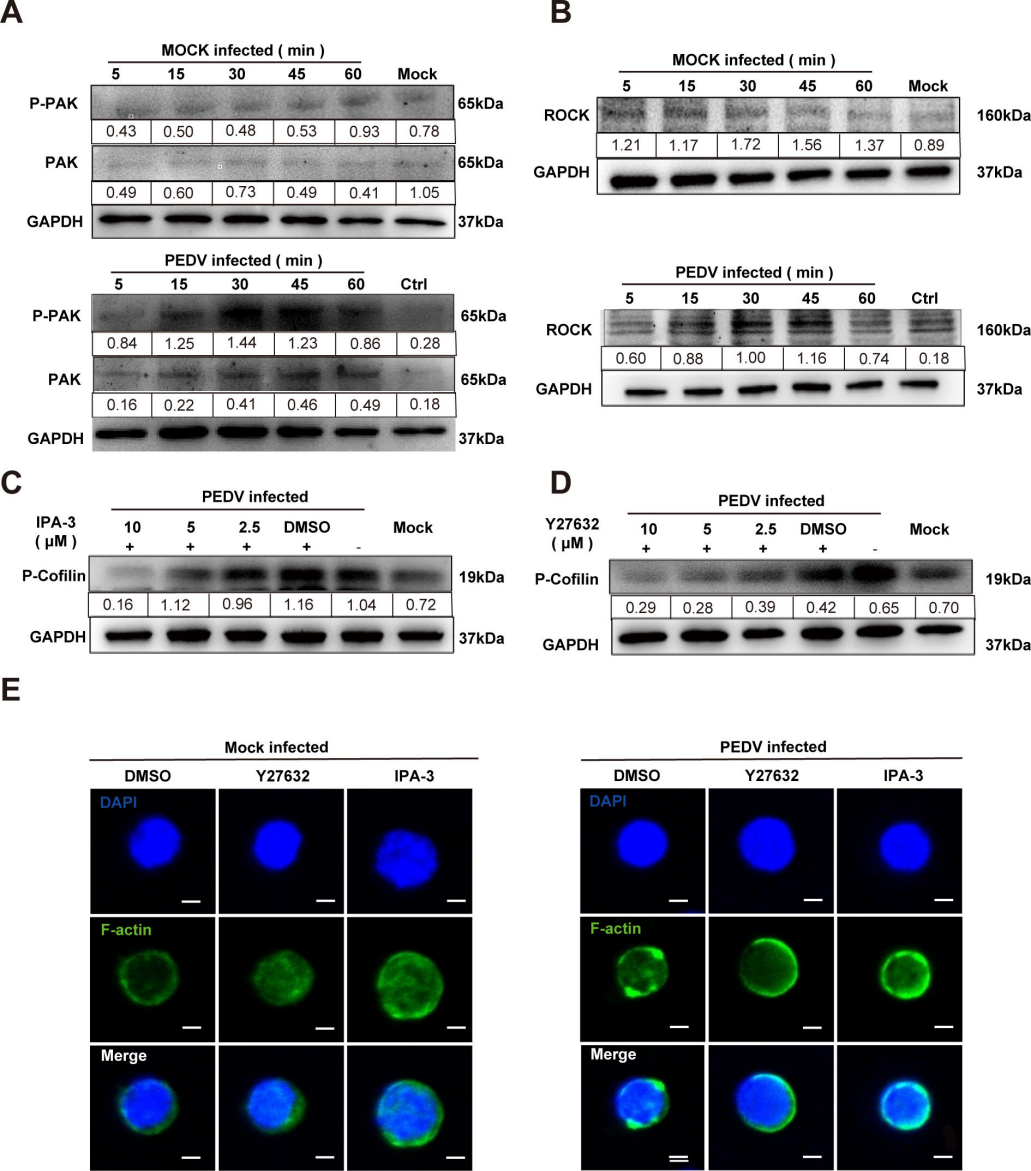
PAK1/ROCK involvement in the regulation of P-cofilin following PEDV invasion. (**A and B**) Activation of PAK and ROCK during PEDV infection. CD4^+^ T cells were inoculated with PEDV (MOI of 1), then cells were collected at indicated times. Lysates of PEDV-infected cells were collected, and the expression of total or phosphorylated proteins of PAK (**A**) and ROCK (**B**) were detected by western blotting. (**C–E**) CD4^+^ T cells were treated with different concentrations of inhibitors and then infected with PEDV effectively inhibited PEDV infection-induced cofilin phosphorylation in CD4^+^ T cells. After treatment with PAK (**C**) and ROCK (**D**) inhibitors, the expression of the phosphorylated protein of cofilin was detected by western blotting, and (**E**) alterations in the cellular microfilament cytoskeleton (green) were evaluated via CLSM. Cell nuclei were stained with DAPI (blue) (scale = 2 µm).

To investigate the potential role of PAK1/ROCK as downstream effectors of Cdc42/Rac1 in the regulation of p-cofilin post-PEDV invasion, the expression levels of PAK1 and ROCK were assessed during PEDV infection. Western blotting analysis revealed significant activation of PAK1 and ROCK in the initial stages of viral infection (Fig. 8A and B). Furthermore, the impact of the PAK inhibitor IPA-3 and the ROCK inhibitor Y-27632 on cofilin phosphorylation was examined. Both IPA-3 and Y-27632 inhibited cofilin phosphorylation in a concentration-dependent manner (Fig. 8C and D). Confocal microscopy analysis further demonstrated that IPA-3 and Y-27632 reversed the alterations in the microfilament cytoskeleton induced by PEDV infection, as illustrated in Fig. 8E.

## DISCUSSION

**Fig 9:**
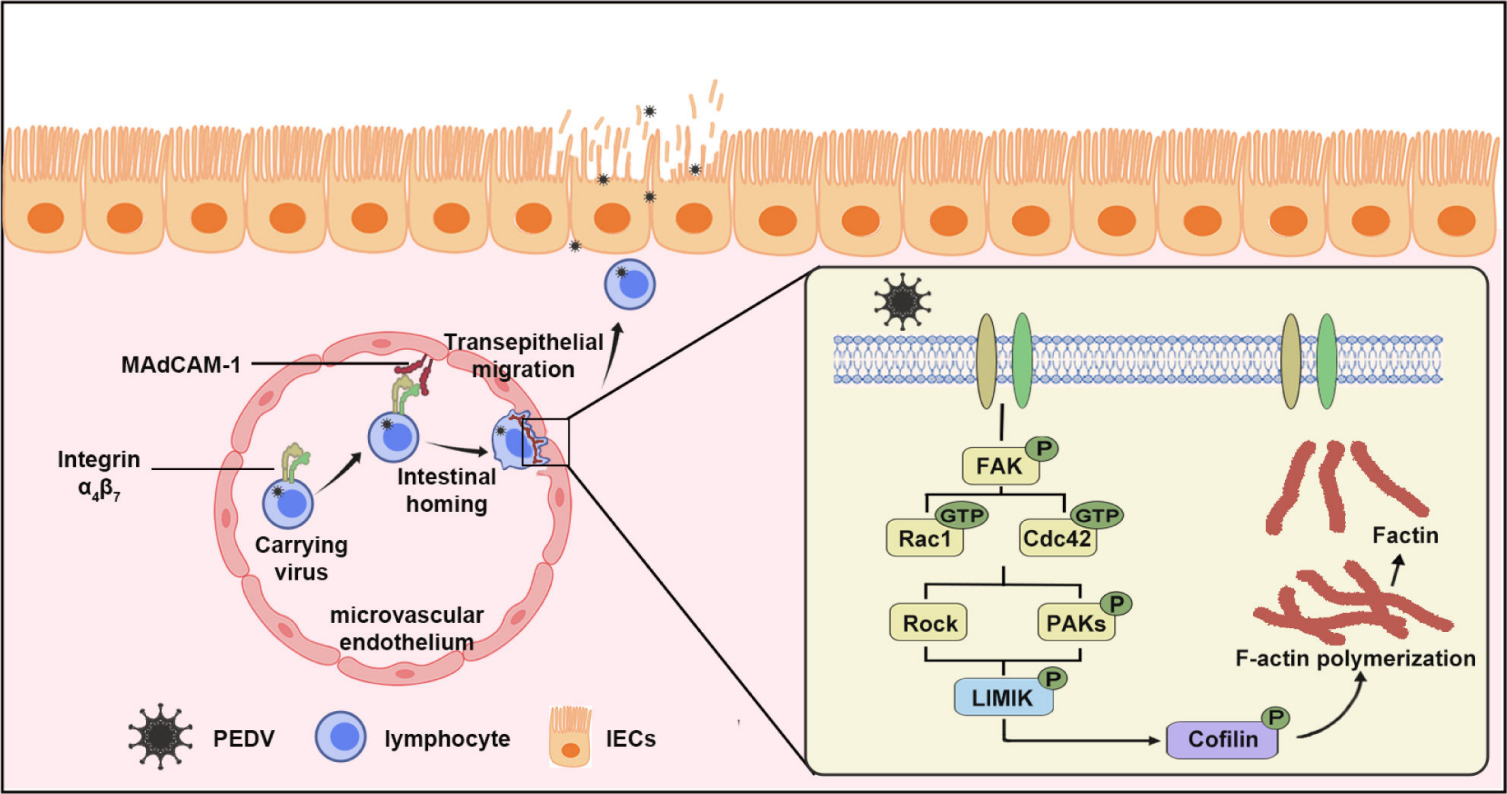
The schematic diagram of the molecular mechanism of PEDV hijacking CD4^+^ T cells for migration to the intestinal mucosa. In previous studies, we have preliminarily elucidated that PEDV can be transmitted through the air and invade nasal mucosa by utilizing submucosal DCs. Subsequently, PEDV-carrying DCs allow the virus to be transferred to peripheral blood T cells via the virological synapse. In our present study, we discovered that the virus primarily enters CD4^+^ T cells. Afterward, PEDV establishes latent infection in CD4^+^ T cells and promotes their homing to the small intestine by upregulating the expression of the small intestinal homing receptor integrin α4β7 on these cells. Furthermore, the virus induces microfilament skeleton rearrangement in CD4^+^ T cells by activating the integrin α4β7-Rho-GTPases-Cofilin signaling pathway, facilitating the formation of cellular pseudopodia. This allows the cells to migrate through post-capillary venules in lymphatic tissue to the lamina propria of the intestinal mucosa and ultimately delivers the virus to intestinal epithelial cells.

PEDV is characterized by its extensive prevalence and rapid transmission, primarily attributed to its aerosol-mediated spread (23, 24). Our prior investigations revealed that upon entering the nasal mucosa via airborne transmission, PEDV is efficiently captured by dendritic cells through transepithelial uptake, a process by which these dendritic cells subsequently transfer intact viral particles to CD4^+^ T cells. Following this transfer, the virus-laden CD4^+^-T cells migrate through the bloodstream, ultimately reaching the small intestinal mucosa and facilitating the infection of intestinal epithelial cells (5). Despite these findings, the precise mechanisms by which PEDV orchestrates the *in vivo* migration and homing of CD4^+^ T cells remain largely unclear and warrant further investigation. To elucidate these mechanisms, we developed *in vivo* and *in vitro* models through which our findings revealed that PEDV establishes latent infection in CD4^+^ T cells and then promotes intestinal homing of CD4^+^ T cells by modulating integrin expression. Therefore, integrins may be a critical factor for PEDV to promote cell adhesion and migration (7, 21). Furthermore, we revealed the intricate molecular pathway by which PEDV induces microfilament rearrangement in CD4^+^ T cells. This process facilitates the formation of cellular pseudopodia, enabling these cells to traverse post-capillary venules within lymphoid tissues, thereby migrating to the intestinal mucosa and ultimately contributing to intestinal pathogenesis (Fig. 9).

Recent research indicates that certain viruses can exploit the *in vivo* migration of immune cells to establish or enhance infections. For instance, VZV(varicella-zoster virus, VZV) can infect peripheral blood lymphocytes, facilitating their migration to the gut and subsequently mediating VZV infection of enteric neurons (22). Similarly, measles virus (MV) infects peripheral blood B lymphocytes, which then migrate back to upper respiratory tract epithelial cells, promoting the spread of the virus from the bloodstream to the respiratory tract (25). In this study, we found that PEDV enters CD4^+^ T cells and promotes their migration to the lamina propria of the small intestine mucosa. This migration ultimately mediates the infection of intestinal epithelial cells by the virus. Thus, the migration of PEDV-carrying CD4^+^ T cells is crucial for the transport of the virus from nonsusceptible sites (the respiratory mucosa) to infection sites (the intestinal mucosa) and its spread from infection sites (the intestinal mucosa) to excretion sites (the oral cavity, nasal cavity, mammary gland, and seminal gland).

Further investigations revealed that PEDV predominantly infects CD4^+^ T-cell subsets, establishing latent infections within them. Although CD4^+^ T cells act as key regulators in the immune system and oversee the activities of various immune cells, including CD8^+^ T cells, their surfaces also express high levels of receptors for certain viruses, allowing these viruses to invade and infect CD4^+^ T cells, thereby allowing them to evade immune surveillance. For instance, HIV promotes viral infection and transmission by binding specifically to CD4 molecules and the coreceptors CCR5 or CXCR4, facilitating the fusion of viral proteins with the cell membrane (26, 27). Additionally, the hepatitis C virus can infect CD4^+^ T cells via receptors such as CD81, SR-BI, and Claudin-1, leading to decreased cell proliferation, enhanced Fas-mediated apoptosis, and inhibited interferon secretion (28, 29). These mechanisms help the virus evade immune surveillance and maintain persistent infection. The possibility that similar receptor molecules on CD4^+^ T cells enable PEDV invasion and the establishment of latent infections requires further investigation. Our study also revealed inconsistencies in the transcription levels of PEDV subgenomic mRNA, with the M gene degrading earlier and more extensively than the N gene. In our experiments, we observed that PEDV invaded CD4^+^ T cells within 1 h post-infection and began transcription approximately 6 h later. The mRNA levels of structural genes essentially reflect the balance between the transcription and degradation processes of viral subgenomic mRNA. Studies have shown that the IE genes (immediate early genes, IE) of HCMV and PRV are downregulated during latent infection. When the IE180 gene is silenced, the transcription of IE180-mediated E (early, E) and L (late, L) genes is restricted, while the transcriptional activity of the LAT gene is enhanced. Therefore, there may be certain similar or complementary regulatory mechanisms within cells that affect the latent state of viral infection (30, 31). Additionally, we found that during the early stages of infection (1–6 h), PEDV effectively inhibited the secretion of IFN-γ and IL-6, while significantly reducing the secretion levels of the anti-inflammatory factor IL-10. However, as the infection progresses, the virus gradually enters a latent infection state after 12 h, at which point the cytokine secretion levels of CD4^+^ T cells gradually return to near-normal baseline. Studies have shown that the IFN-α/β-induced pathway is crucial for the quiescent state during the latency of gamma herpesvirus, yet over time, the IFN-α/β pathway becomes dispensable (32). This indicates that there is a dynamic change in the expression between viral genes and interferon genes during latent infection, and there is a continuous arms race between the expression of viral mRNA or viral proteins and the expression of host innate immune response genes during infection and latency (33).

After homing to intestinal lymphoid tissue, virus-expressing CD4^+^ T cells need to traverse post-capillary venules to further migrate into the intestinal mucosal lamina propria. During this process, lymphocytes initially utilize surface adhesion molecules such as LFA-1 and VLA-4 to bind to corresponding ligands on endothelial cells, facilitating “rolling” along vessel walls (34). As rolling continues, lymphocytes stabilize their interaction through integrin molecules on their surface binding to ICAM-1 and VCAM-1 on endothelial cells, transitioning into the “firm adhesion” phase (35). This firm adhesion is reinforced by “inside-out” signaling, which enhances lymphocyte polarization and migration capabilities. Integrin α4β7, a crucial gut-homing receptor on CD4^+^ T cells, interacts with MAdCAM-1 to facilitate the rolling and stable adhesion of circulating lymphocytes to the vascular endothelium of the intestinal mucosa (36, 37). This interaction enables lymphocytes to directionally migrate to the gut, where they participate in immune responses and inflammatory processes. Research indicates that viruses can manipulate integrins on immune cells to enhance their dissemination and infection within the host. For instance, cytomegalovirus employs integrin αVβ3 as a coreceptor to invade monocytes/macrophages, activating intracellular signaling pathways such as the PI3K pathway, promoting endocytosis, and accelerating viral infection (38). Similarly, the envelope glycoprotein gp120 of HIV-1 interacts with integrin α4β7 on CD4^+^ T cells, enhancing viral binding and entry and thereby facilitating its dissemination within the host (39). Our study demonstrated that PEDV infection significantly upregulates integrin α4β7 expression on CD4^+^ T cells. Inhibiting this integrin effectively prevents the migration of these infected lymphocytes into the gut, thereby blocking infection. This finding suggested that integrin α4β7 plays a significant role in the gut-homing mechanism of CD4^+^ T cells during PEDV infection and could be targeted to prevent PEDV-induced intestinal infections originating from respiratory routes.

Furthermore, CD4^+^ T cells traverse the tight junctions of endothelial cells by forming invasive pseudopodia, completing the critical step of migration from the bloodstream to the intestinal mucosal lamina propria. During this migration, CD4^+^ T cells develop lamellipodia at their leading edge, with a thickness of 0.1–0.2 μm, enabling the cells to extend forward and navigate through the gaps between endothelial cells and the extracellular matrix (40). Our study revealed that PEDV infection induces the formation of lamellipodia on the surface of CD4^+^ T cells within 30 min, accompanied by significant rearrangement of the actin cytoskeleton. The actin cytoskeleton, which is composed of a complex network of actin filaments, plays a crucial role in maintaining cell morphology, motility, and division (41, 42). Previous research has established that the rearrangement of the actin cytoskeleton underpins the formation and dynamic extension of invasive pseudopodia, which involves cycles of actin polymerization and depolymerization (43). Specifically, actin monomers (G-actin) polymerize into actin filaments (F-actin) at the cell’s leading edge, promoting the extension of pseudopodia. Subsequently, F-actin depolymerizes to release G-actin, providing the raw material for further actin polymerization, thus supporting continuous pseudopodia growth and forward movement. Our findings indicate that pretreatment of lymphocytes with the actin-depolymerizing agent CytoD significantly inhibits the formation of PEDV-induced lamellipodia, suggesting that PEDV facilitates pseudopodia formation by modulating the lymphocyte actin cytoskeleton. Numerous studies have reported that viruses manipulate the actin cytoskeleton of host cells to enhance their infection. For example, rabies virus and HIV promote filopodium formation in host cells by regulating actin filament polymerization, aiding in viral entry and intracellular transport (44, 45). Similarly, influenza A virus infection induces dynamic rearrangement of actin filaments, enhancing cell-cell junction formation and viral spread to adjacent cells (46). Our previous studies have shown that PEDV induces actin cytoskeleton remodeling in intestinal epithelial cells, facilitating viral replication and release. Therefore, in-depth research into the interaction between PEDV and the host cell actin cytoskeleton may lead to the development of novel antiviral strategies targeting the cytoskeleton.

While numerous studies have reported that classical signaling pathways such as the PI3K/Akt, MAPK, and TGF-β pathways are involved in regulating the actin cytoskeleton (47, 48), our findings underscore the pivotal role of the Rho-GTPase family in PEDV-mediated regulation of the actin cytoskeleton in CD4^+^ T cells. The Rho-GTPase family acts as a fundamental molecular switch for the actin cytoskeleton and comprises several small GTPases, including Cdc42-GTPase, Rac1-GTPase, and RhoA-GTPase, which regulate actin organization and cellular morphology (49). In our study, we found that Rac1-GTPase and Cdc42-GTPase are crucial for the activation of cofilin and the rearrangement of the actin cytoskeleton following PEDV infection in CD4^+^ T cells. The regulation of the actin cytoskeleton by members of the Rho GTPase family hinges on the activation of downstream actin-depolymerizing factors, among which cofilin plays a critical role in stabilizing and depolymerizing the actin cytoskeleton. Under normal conditions, cofilin binds preferentially near the ADP-binding site of actin filaments, enhancing actin severing and depolymerization. When phosphorylated and inactivated, cofilin promotes actin polymerization. This regulatory mechanism facilitates dynamic remodeling of the actin cytoskeleton, aiding in the formation and migration of lymphocyte pseudopodia (50, 51). Our study demonstrated that PEDV tightly regulates the activity of cofilin in CD4^+^ T cells. Specifically, 30 min post-infection, cofilin phosphorylation levels increase, leading to cofilin inactivation. Conversely, 1 h post-infection, cofilin phosphorylation decreased, restoring its activity. These dynamic changes in cofilin activity are closely correlated with PEDV-induced actin cytoskeleton rearrangement in CD4^+^ T cells. These findings reveal the critical role of the Rho-GTPase family in modulating dynamic changes in the actin cytoskeleton during PEDV infection.

To summarize, this study explains how PEDV infection through the nasal mucosa causes intestinal pathogenesis in piglets by promoting the movement of CD4^+^ T cells from the peripheral circulation. Our results indicated that PEDV mostly induces dormant infections in CD4^+^ T cells and increases the expression of integrin α4β7, which in turn enhances their migration to the gut. Furthermore, we determined the specific molecular process through which PEDV triggers the activation of the integrin α4β7-mediated Rho-GTPase-Cofilin signaling pathway. This system is responsible for controlling the development of pseudopodia in CD4^+^ T cells, enabling their movement toward the lamina propria of the intestinal mucosa. These discoveries offer encouraging opportunities for devising efficient methods to manage and prevent PEDs. Additionally, our analysis provides valuable reference points for investigating the disease-causing processes of other viruses that share comparable infection features, potentially facilitating future research and therapeutic strategies.

## MATERIALS AND METHODS

### Cells and viruses

Vero E6 cells were kindly provided by the Veterinary Medicine Research Center of the Da Bei Nong Group. IPEC-DQ was purchased from Jennio Biotech Company in Guangzhou, China. The Vero E6 and IPEC-DQ cell lines were cultured in Dulbecco’s modified Eagle medium (DMEM) supplemented with 10% fetal bovine serum (FBS) and 100 U/mL penicillin and streptomycin. The wild-type PEDV strain Zhejiang08, which was clustered with the emerging virulent strain, was preserved by our laboratory.

### Antibodies, small-compound inhibitors, and siRNAs

Rabbit anti-cofilin (EPR6375) and anti-phospho-cofilin Mabs (EPR24753-28) were purchased from Abcam (Cambridge, UK). Rabbit anti-Rho-GTPase Mab (9968), anti-PAK1 PAb (2602), and anti-phospho-PAK1 PAb (2601) were purchased from Cell Signaling Technology (Beverly, MA). Rabbit anti-phospho-FAK PAb (44-624G) was purchased from Thermo Fisher Scientific (USA). Rabbit anti-integrin-α4β7 Rab was purchased from BIOSS (Beijing, China). The anti-PEDV N protein MAb (SD17-103) was purchased from Medgene Labs (Vermillion, USA). FITC-conjugated integrin beta 7 MAb (MA5-23592) was purchased from Invitrogen (MA, USA). FITC-phalloidin (40735ES75) was purchased from Yeasen (Shanghai, China). CCG-1423 (B4897), PF-573228 (B1523), and EHoP-016 (B2219) were purchased from APExBIO (Houston, USA). CT7758 (HY-70073), Y-27632 (HY-10071), ML-141 (HY-12755), and IPA3 (HY-15663) were purchased from MCE (New Jersey, USA). Mouse anti-CD4 antibody was purchased from BD Pharmingen (New Jersey, USA). The protein marker (GF6166) was purchased from Genefist (Shanghai, China). The cofilin siRNA primers used are listed in Table S2.

### *In vivo* competition experiments

The *in vivo* competition assays were performed as described previously in our laboratory. PBMCs were isolated from the blood of three littermate piglets that were either treated or not treated with PEDV. These PBMCs were then purified to get T lymphocyte cells and labeled with CFSE (green) or CM-DiD (red, from Molecular Probes) for long-term cell labeling. CM-DiD-labeled T lymphocyte cells were further treated with the inhibitor CT7758. Equal numbers of cells (1  ×  10^7^) from the two populations were then mixed and adoptively transferred into piglets (autologous) via front cavity vein injection. The recipient piglets were sacrificed 24 h after the injection. For flow cytometry analyses, cells were isolated from MLN and the jejunum.

Piglets were randomly assigned to either an inhibitor group or a PEDV-infected group, with three piglets in each group. T lymphocyte cells were isolated and purified from the piglets in both groups. T lymphocyte cells of the inhibitor group were further treated with the inhibitor CT7758. Subsequently, both groups of piglets were administered PEDV treatment and adaptively transferred into recipient piglets (autologous) via front cavity vein injection. The recipient piglets were euthanized 24 h after injection. For histological examination, the jejunum tissue was fixed with 4% formaldehyde and embedded in paraffin. The tissue sections were then inoculated with an anti-PEDV-N antibody (provided by Median Diagnostics) to assess the levels of PEDV through immunohistochemical and immunofluorescence staining.

### Generation of CD4^+^ T cells

Using a pig peripheral blood lymphocyte isolation kit (TBD), PBMCs were successfully isolated from pig blood through density centrifugation. The following method was used to isolate MLN lymphocytes: the lymph nodes were rinsed three times in PBS containing 2% penicillin/streptomycin, 1% gentamicin, and 1% cephalosporin. To obtain CD4^+^ T cells, PBMC lymphocytes were labeled with APC-CD4 antibodies, coated with anti-FITC microbeads, and sorted using the MiniMACS starting kit. The sorted CD4^+^ T cells were then activated with PHA and IL-2 (Proceth) to prepare for subsequent experiments.

### *In vitro* T-cell migration assay

First, the matrix gel was thawed and diluted with a serum-free medium at a ratio of 1:5 according to the original solution. The bottom membrane of the Transwell chamber was coated with the diluted matrix gel and inoculated at 37°C for 30 min to allow the matrix gel to polymerize into a gel. Before use, the base membrane was hydrated. The peripheral blood-derived T cells were adjusted to a density of 2 × 10^5^ cells/mL with serum-free medium containing BSA, labeled with CFSE, infected with PEDV, and seeded onto Matrigel. In the lower chamber of a 24-well plate, 600 µL of culture medium containing MAdCAM-1 (500 ng/mL) was added, avoiding the formation of bubbles between the culture medium and the chamber. Bubbles can weaken or even eliminate the chemotactic effect of the lower chamber. The cells were then inoculated at 37°C with 5% CO_2_ for 24 h. Finally, the chamber was placed on a slide and observed under a Nikon A1 confocal microscope.

### Transfer infection

IPEC-DQ cells were seeded in 24-well plates and cultivated into monolayers prior to infection. CD4^+^ T cells harboring PEDV were cocultured with IPEC-DQ cells via two methods: direct contact and indirect contact. In the contact method, CD4^+^ T cells were directly cultured with IPEC-DQ cells, while in the noncontact method, CD4^+^ T cells were grown in Transwell assemblies with a pore diameter of 0.4 µm above the IPEC-DQ cell monolayers. To verify that cell-free infectious PEDV could pass through the filters in the Transwell assemblies, negative controls (uninfected CD4^+^ T cells) and positive controls (consisting of an identical number of PEDV viruses placed over 0.4 µm filters) were used. The medium from the CD4^+^ T cell and IPEC-DQ cell cocultures was collected at the designated time points. To eliminate nonabsorbed viruses and CD4^+^ T cells, IPEC-DQ cells were washed five times with PBS and subsequently cultured in a maintenance medium for 96 h.

### Plaque assay

After Vero E6 cells were seeded into a 12-well plate and allowed to form a monolayer under culture conditions of 37°C and 5% CO_2_, the cells were inoculated with virus stock (1 mL of serial 10-fold dilutions) and inoculated for 1 h. Subsequently, the cells were overlaid with 0.7% low-melting-point agarose in DMEM containing 2% FBS and inoculated at 37°C for approximately 72 h. To visualize plaques, the cells were finally stained by soaking in a methanol solution containing 1% crystal violet.

### RNA sequencing

Total RNA from mock-infected, PEDV-infected, and inhibitor-pretreated groups were extracted using TRIzol Reagent (Invitrogen, USA). After testing the quality and integrity, 2 µg of total RNA was used for stranded RNA sequencing library preparation using KC Stranded mRNA Library Prep Kit for Illumina (Catalog No. DR08402, Wuhan Seqhealth Co., Ltd. China). Next, the mRNA libraries were constructed and sequenced using a DNBSEQ-T7 sequencer (MGI Tech Co., Ltd. China) with a PE150 model.

### Western blotting

At the indicated times post-infection, the cells were washed with PBS and lysed in ice-cold cell lysis buffer containing protease and phosphatase inhibitors. Equal amounts of protein were separated by SDS-PAGE and transferred to a PVDF membrane. The membrane was blocked with 5% BSA in Tris-buffered saline containing 0.1% Tween 20, inoculated overnight at 4°C with primary antibodies (1:1,000), rinsed and inoculated with the corresponding HRP-conjugated secondary antibodies (1:5,000) for 1 h at 37°C. Antibody binding was detected by autoradiography using ECL. Western blotting was quantified by Quantity One (Quantity One 1-D Analysis Software 170-9600, Bio-Rad). The intensity of the bands in terms of density was measured and normalized to that of GAPDH. All the data are expressed as the means ± SDs of three independent experiments.

### RNA isolation and qRT-PCR

Total RNA was extracted using TRIzol (Invitrogen) according to the manufacturer’s manual. The samples were dissolved in RNase-free water, digested with DNase I, and then reverse transcribed using the HiScript III 1st Strand cDNA synthesis kit (Vazyme). Quantitative RT-PCR was performed using a TB Green qPCR kit (TaKaRa) on an Applied Biosystems 7500 fast real-time PCR system (Life Technologies). The GAPDH gene was used as the reference gene for normalization. All primers used for RT-qPCR are listed in Table S1.

### Immunofluorescence assays and confocal microscopy

CD4^+^ T cells were inoculated with PEDV at 4°C for 1 h and then transferred to a 37°C environment. At the designated time points, the cells were washed with PBS and fixed with 4% formaldehyde. FITC-phalloidin was added to the samples for 30 min to stain the cell skeleton, and the samples were then washed with PBS and inoculated with primary antibody (1:100) at 4°C overnight. After the cells were washed, they were inoculated with a fluorescently labeled secondary antibody (1:200) for 30 min at room temperature, washed three times again with PBS, and then inoculated with 1 µg/mL DAPI for 5 min. Images were captured using a Nikon A1 confocal microscope (Nikon, Japan).

### Statistical analysis

The results are presented as the means ± SDs and were analyzed with SPSS 17.0. One-way analysis of variance (ANOVA) was used to determine significant differences among multiple groups, and a *t*-test was used to determine differences between two groups. *P < 0.05, **P < 0.01. The data were combined from at least three independent experiments unless otherwise stated. The sample size is indicated for each experiment in the corresponding figure legend.

## Data Availability

The authors declare that all data supporting the findings of this study are available in the manuscript and its Supplementary Information files or are available from the corresponding author upon request. Raw data underlying the plots in the figures, as well as uncropped western blot images, are available in the supplementary material. The raw data of RNA Sequencing data have been deposited in the GenBank repository under BioProject ID PRJNA1136653.

## SUPPLEMENTAL MATERIAL

Fig. S1 jvi.01761-24-s0001.tif Analysis of transcriptome profiles of CD4+ T cells after infection with PEDV.

Fig. S2 jvi.01761-24-s0002.tif Impact of inhibitors at different concentrations on the proliferation of CD4+ T cells detected by the CCK8 assay.

Fig. S3 jvi.01761-24-s0003.tif Identification of the interference efficacy of cofilin in CD4+ T cells.

Fig. S4 to S8 jvi.01761-24-s0004.docx Uncropped western blots.

Supplemental material jvi.01761-24-s0005.docx Legends for Fig. S1 to S3; Tables S1 to S3.

Supplemental data set jvi.01761-24-s0006.xlsx Raw data for all images.

ASM does not own the copyrights to Supplemental Material that may be linked to, or accessed through, an article. The authors have granted ASM a non-exclusive, world-wide license to publish the Supplemental Material files. Please contact the corresponding author directly for reuse.
