# Supplementary figures and images for: In vivo investigation of PEDV transmission via nasal infection: mechanisms of CD4+ T-cell-mediated intestinal infection
Source: J Virol. 2025 Mar 17;99(4):e01761-24. doi: 10.1128/jvi.01761-24 (PMC12020991; doi:10.1128/jvi.01761-24)

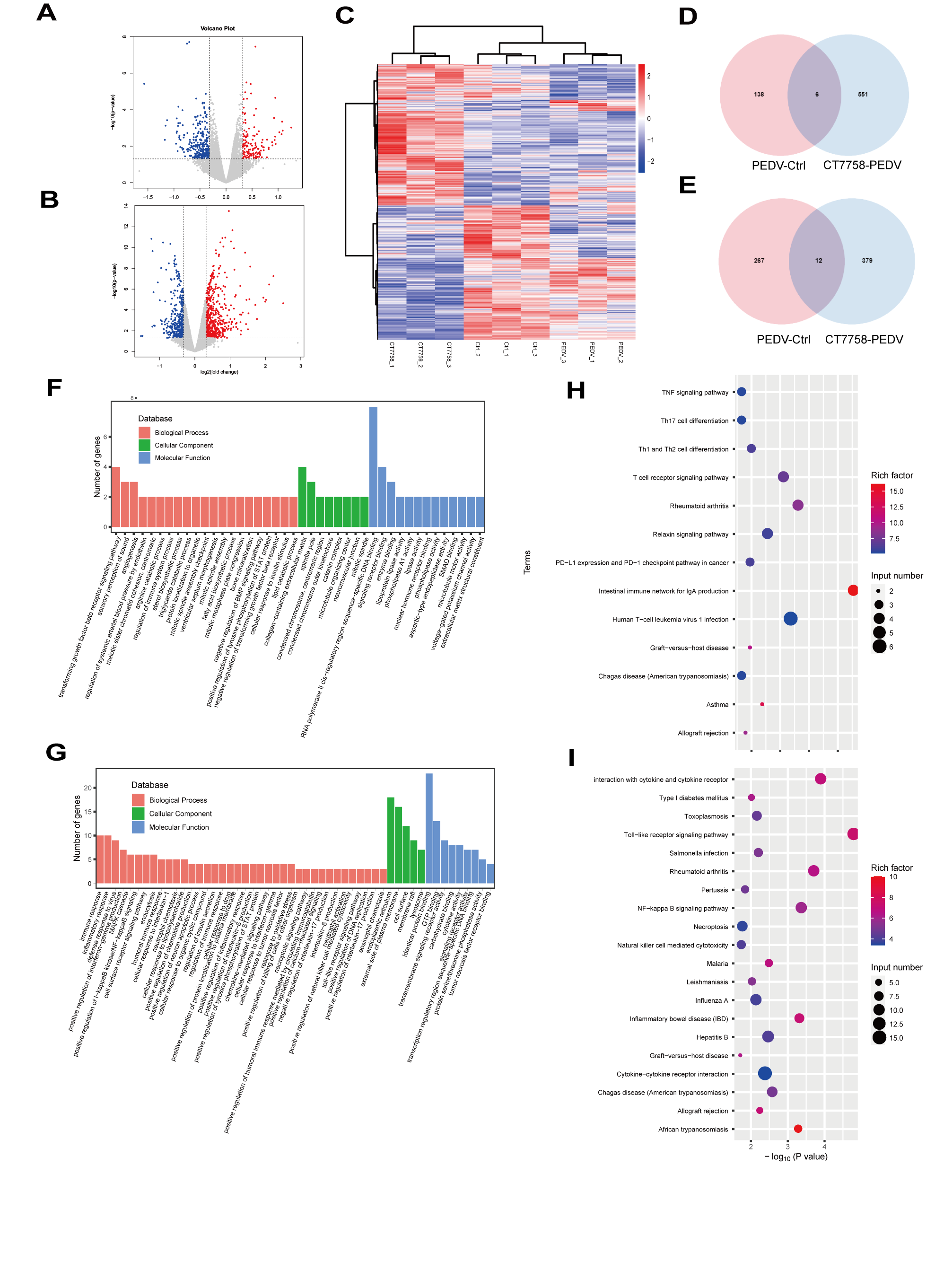

Supplement: Fig. S1 — Analysis of transcriptome profiles of CD4+ T cells after infection with PEDV. [file jvi.01761-24-s0001.tif]

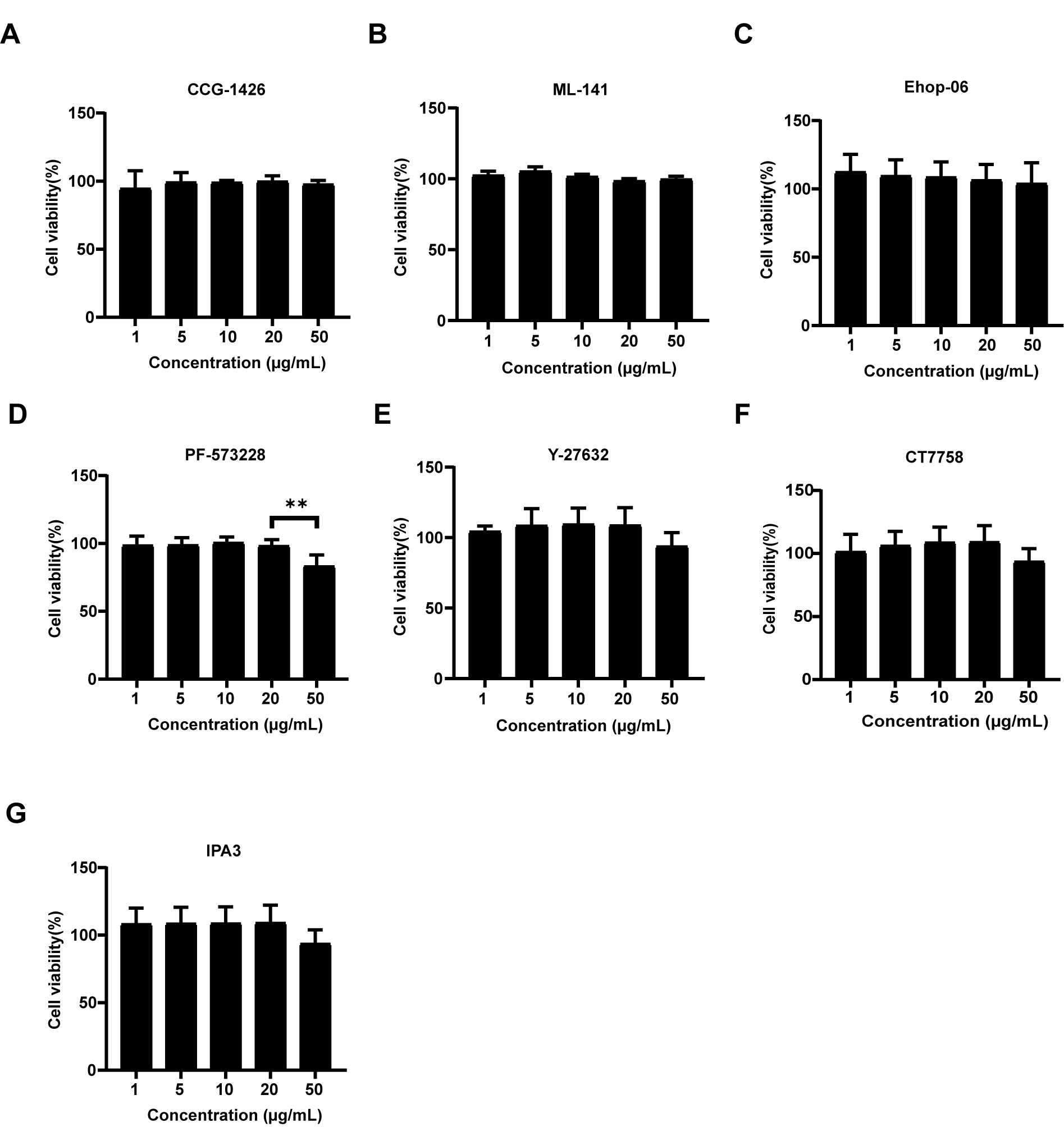

Supplement: Fig. S2 — Impact of inhibitors at different concentrations on the proliferation of CD4+ T cells detected by the CCK8 assay. [file jvi.01761-24-s0002.tif]

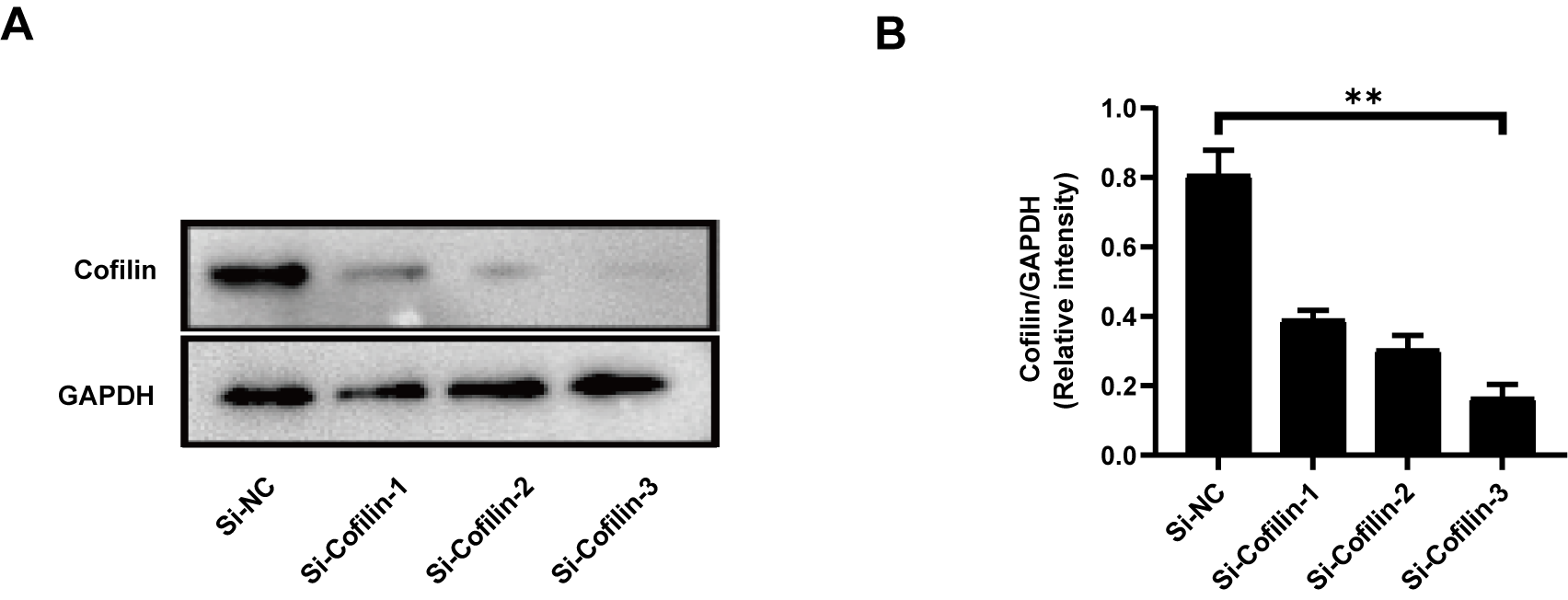

Supplement: Fig. S3 — Identification of the interference efficacy of cofilin in CD4+ T cells. [file jvi.01761-24-s0003.tif]
