# Supplementary material for: In vivo investigation of PEDV transmission via nasal infection: mechanisms of CD4+ T-cell-mediated intestinal infection
Source: J Virol. 2025 Mar 17;99(4):e01761-24. doi: 10.1128/jvi.01761-24 (PMC12020991; doi:10.1128/jvi.01761-24)
Supplement: Fig. S4 to S8 — Uncropped western blots. [file jvi.01761-24-s0004.docx]

**All western blots**

The protein marker (GF6166) was purchased from Genefist (Shanghai, China).


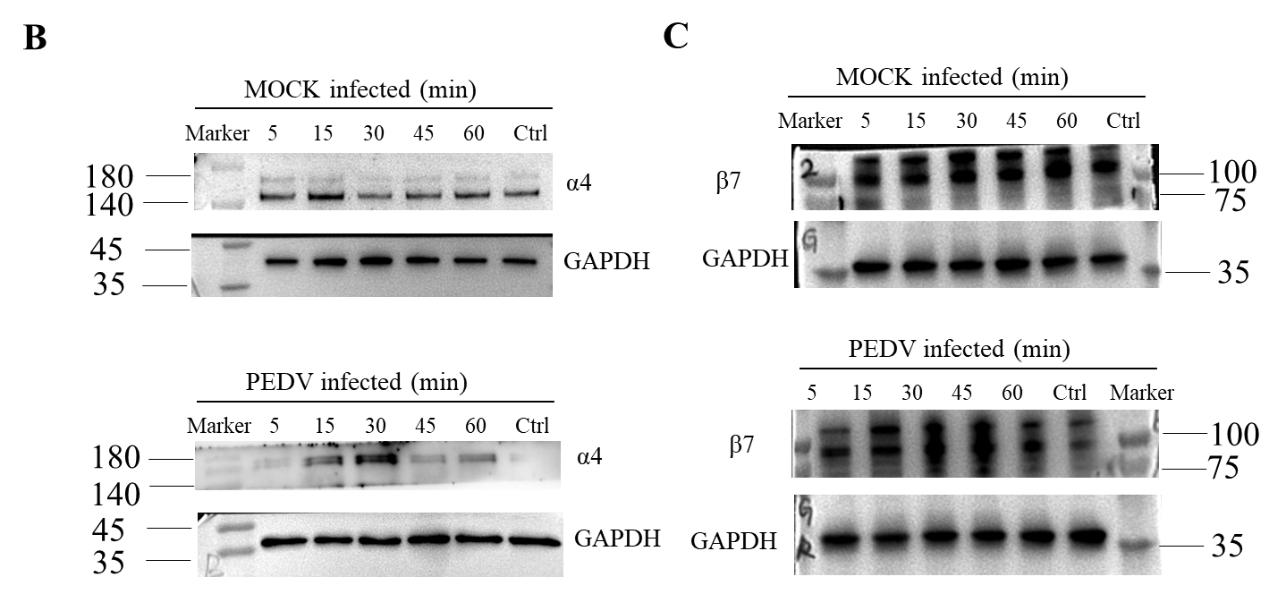


**Supp figure 4.** **Full western blots for Figure 2B, 2C.**


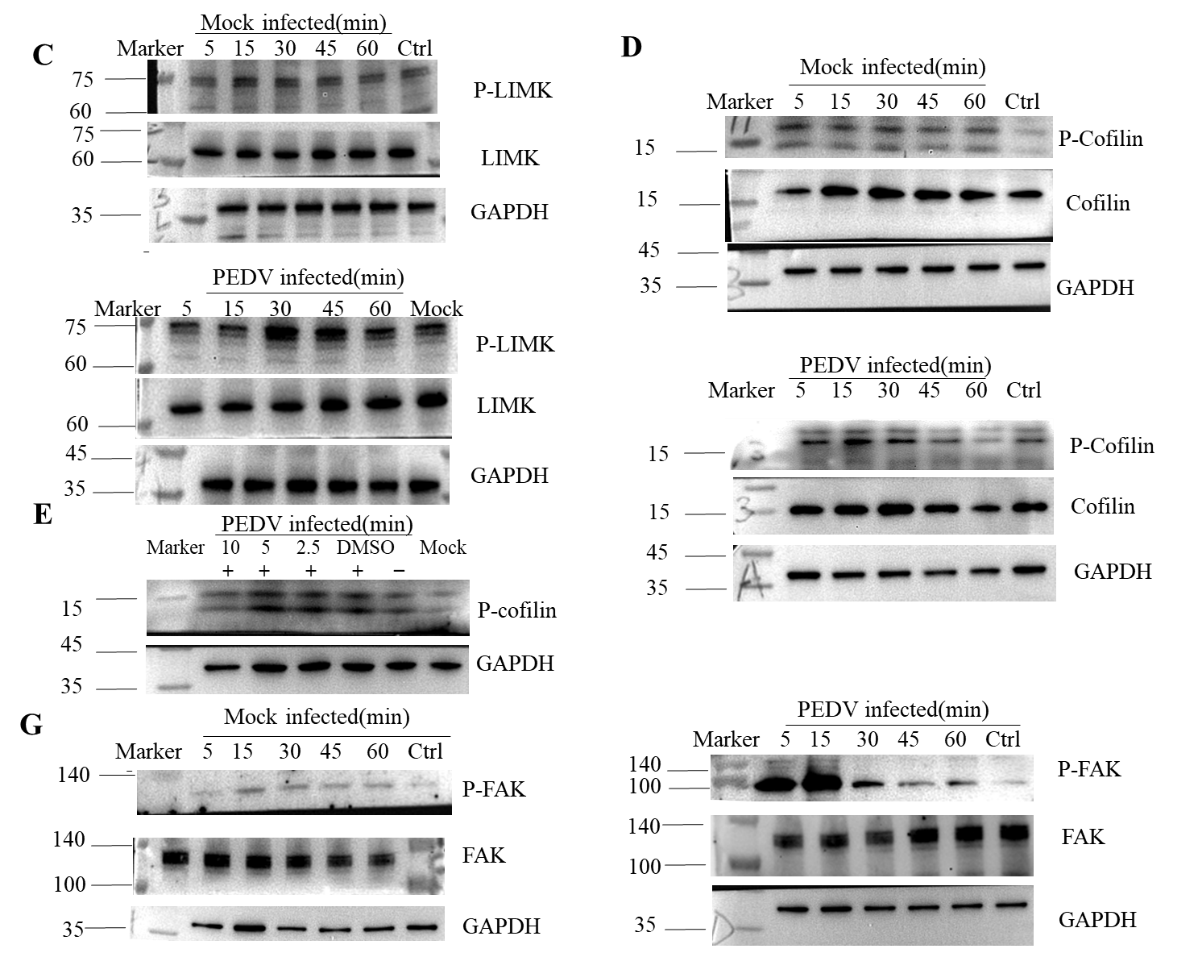


**Supp figure 5.** **Full western blots for Figure 6C, 6D, 6E 6G.**


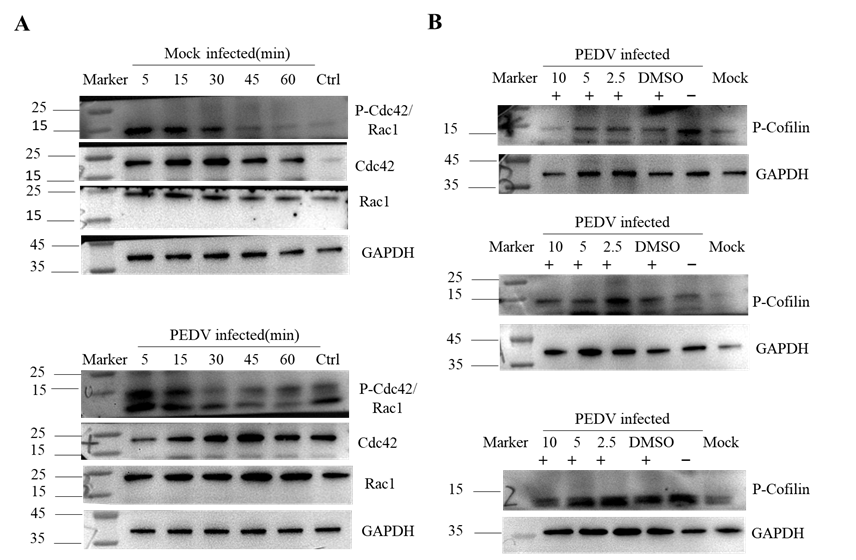


**Supp figure 6.** **Full western blots for Figure 7A, 7B.**

**
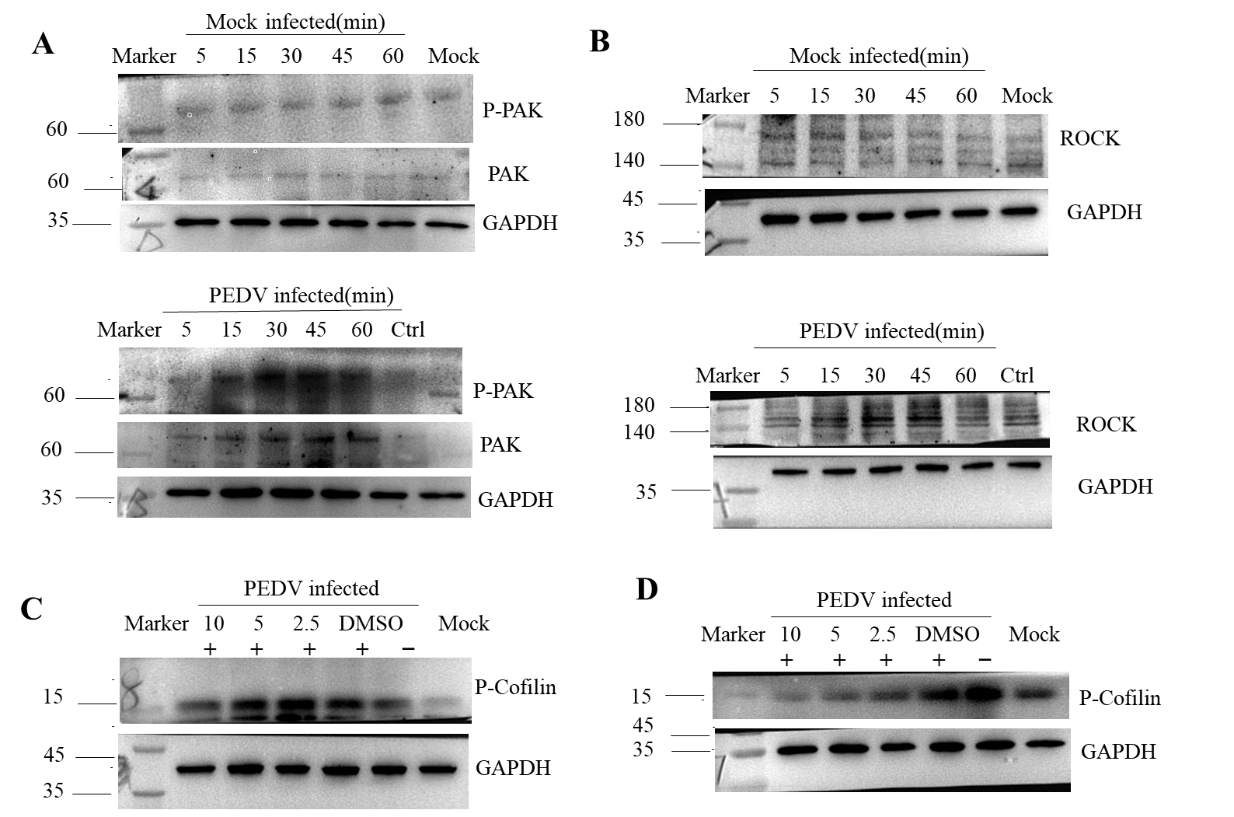
**

**Supp figure 7.** **Full western blots for Figure 8A, 8B, 8C, 8D.**


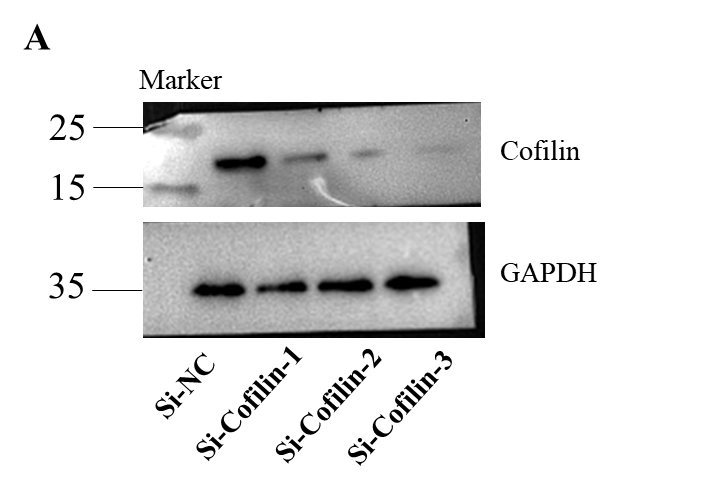


**Supp figure 8.** **Full western blots for Supp figure 3a.**
