# Supplementary material for: In vivo investigation of PEDV transmission via nasal infection: mechanisms of CD4+ T-cell-mediated intestinal infection
Source: J Virol. 2025 Mar 17;99(4):e01761-24. doi: 10.1128/jvi.01761-24 (PMC12020991; doi:10.1128/jvi.01761-24)
Supplement: Supplemental material — Legends for Fig. S1 to S3; Tables S1 to S3. [file jvi.01761-24-s0005.docx]

Supporting Information

Title: *In vivo* investigation of PEDV transmission via nasal infection: mechanisms of CD4^+^T-cell-mediated intestinal infection

**Qiu Zhong^1^, Jiaxin Qi^1^, Na Su^1^, Zi Li^2^, Hui Zeng^1^, Ruiling Liu^1^, Yuchen Li^1^*,**

**Qian Yang^1^***

1 MOE Joint International Research Laboratory of Animal Health and Food Safety, College of Veterinary Medicine, Nanjing Agricultural University, Nanjing, Jiangsu, China

2 State Key Laboratory for Diagnosis and Treatment of Severe Zoonotic Infectious Diseases, Key Laboratory for Zoonosis Research of the Ministry of Education, Institute of Zoonosis, and College of Veterinary Medicine, Jilin University, Changchun 130062, China.

*** Corresponding Authors:**

[yuchenli2022@njau.edu.cn](mailto:yuchenli2022@njau.edu.cncom) (Yuchen Li); [zxbyq@njau.edu.cn](mailto:zxbyq@njau.edu.cn) (Qian Yang)


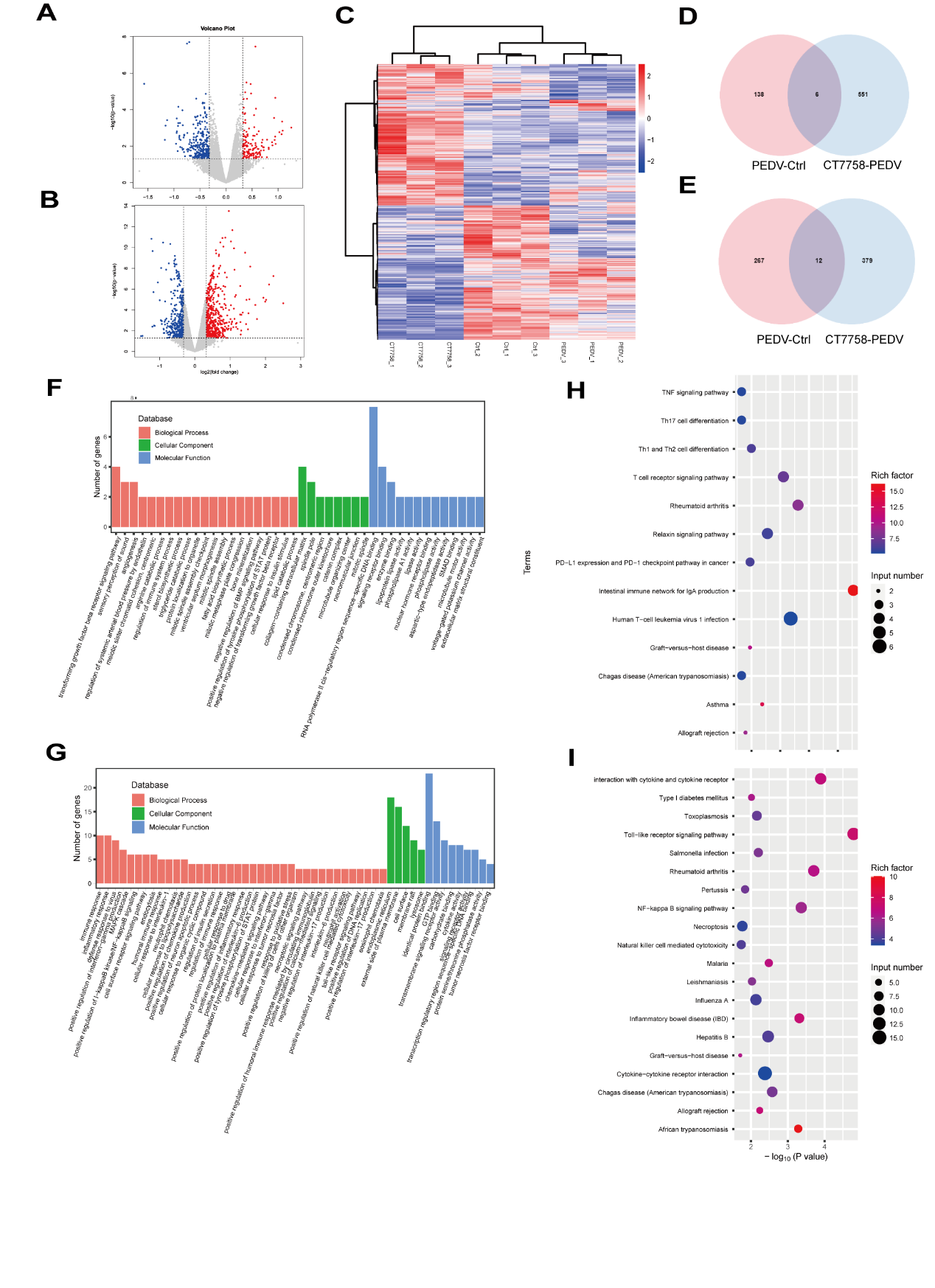
 **Supp figure 1 Analysis of transcriptome profiles of CD4^+^ T cells after infection with PEDV** (A) Volcano plot displaying DEGs between the mock-infected and PEDV-infected groups. (B) Volcano plot illustrating differentially expressed genes (DEGs) between the PEDV-infected and inhibitor-pretreated groups. (C) Cluster analysis of differentially expressed genes (DEGs) across the three groups. (D) Venn diagram showing the overlap of differentially expressed genes (DEGs) between the mock-infected and PEDV-infected groups. (E) Venn diagram depicting the overlap of differentially expressed genes (DEGs) between the PEDV-infected and inhibitor-pretreated groups. (F and G) GO and KEGG enrichment analysis of upregulated differentially expressed genes (DEGs) between the mock-infected and PEDV-infected groups. (H and I) GO and KEGG enrichment analysis of downregulated differentially expressed genes (DEGs) between the PEDV-infected and inhibitor-pretreated groups.


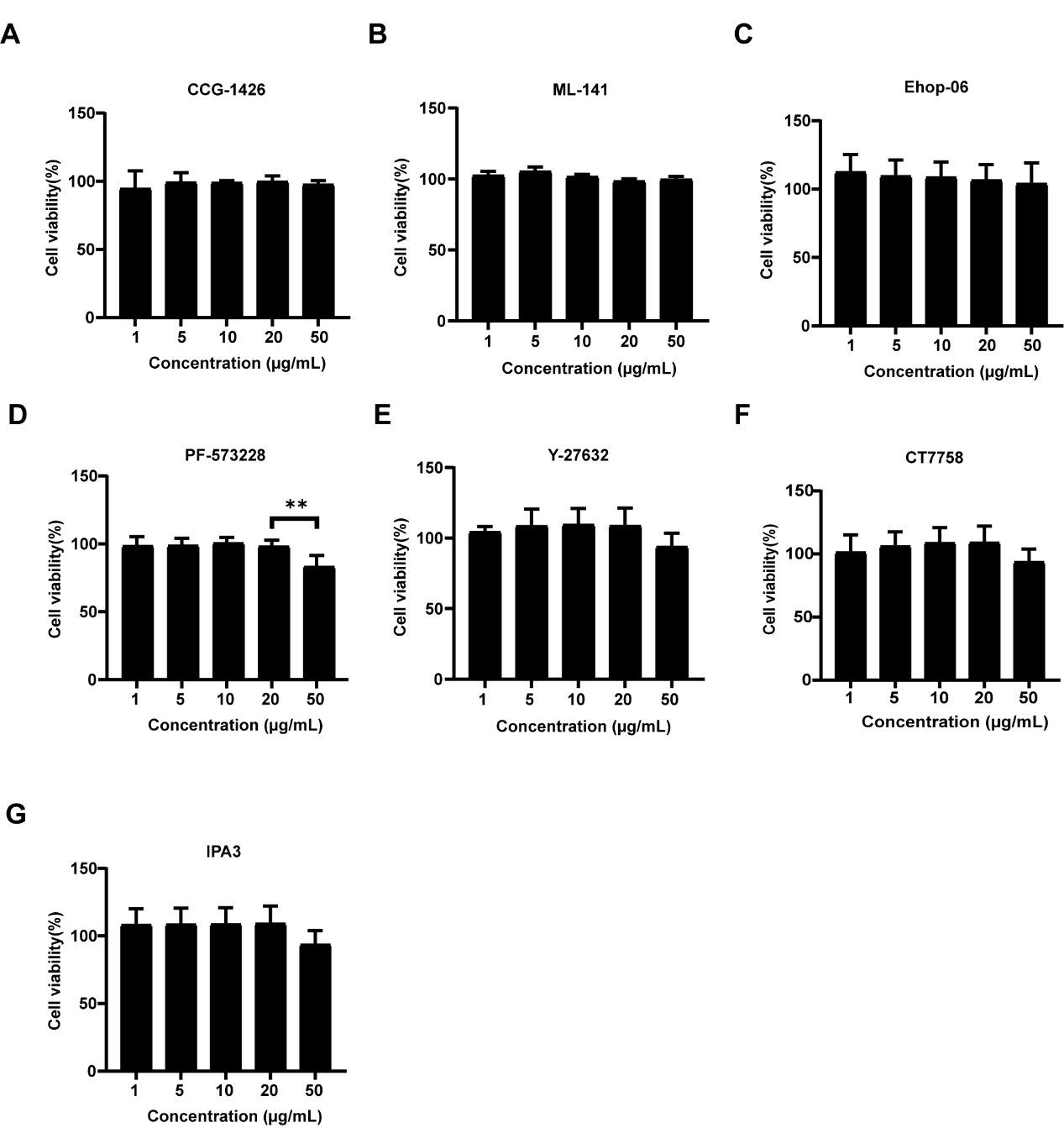


**Supp figure 2 Impact of inhibitors at different concentrations on the proliferation of CD4^+^ T cells detected by the CCK8 assay** CCK8 analysis of CD4^+^ T cells incubated with (A) CCG-1426, (B) ML-141, (C) Ehop-06, (D) PF-573228, (E) Y-27632, (F) CT7758 and (G) IPA-3. All the data are presented as the means ± SDs, and comparisons were performed using one-way ANOVA. **P* < 0.05, ***P* < 0.01. The results are from at least three different experiments. CCK8, cell counting kit 8.


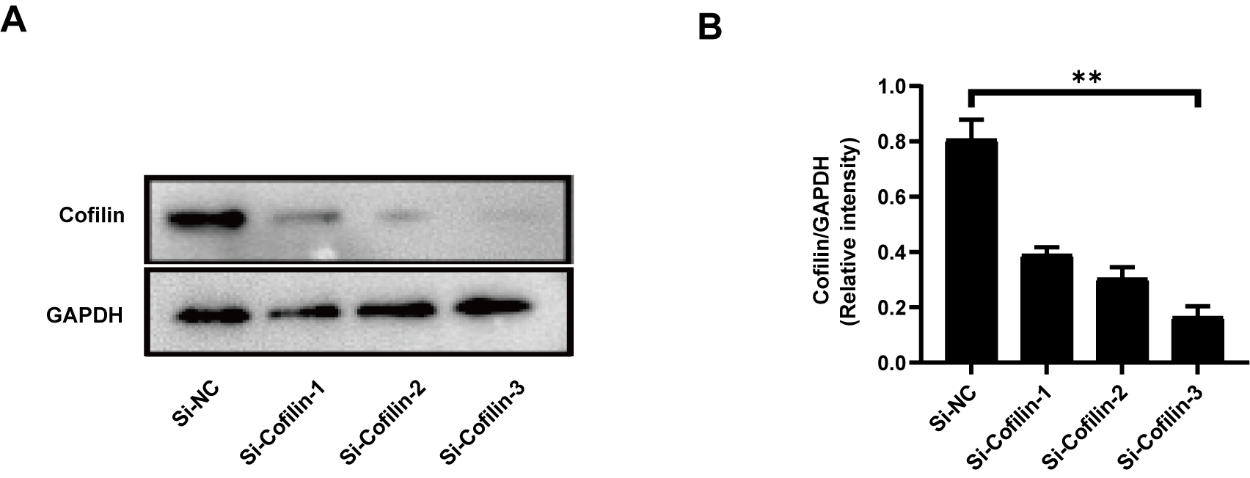


**Supp figure 3 Identification of the interference efficacy of cofilin in CD4^+^ T cells** (A) The efficacy of cofilin silencing was analyzed by western blotting. (B) The bar chart shows the statistical results of western blotting. All the data are presented as the means ± SDs, and comparisons were performed using one-way ANOVA. **P* < 0.05, ***P* < 0.01. The results are from at least three different experiments.

**Supp table 1 Ct values of PEDV non-structural genes**

| Genes | Primers | Sequence (5'-3')* |
| --- | --- | --- |
| Integin α4  (Sus scrofa) | Forward | CATTGGTGAGCAGGCAATGT |
|  | Reverse | TGATGAGCTTCTCCTGCCTTG |
| Integin β7  (Sus scrofa) | Forward | CCCGGTGCCAAGGATTACAAG |
|  | Reverse | AGATCAGGACTCCCCCGTT |
| GAPDH  (Sus scrofa) | Forward | ACATCATCCCTGCCTCTACTG |
|  | Reverse | CCTGCTTCACCACCTTCTTG |

**Supp table 2 SiRNA sequences used for RNA interference**

| Genes | Primers | Sequence (5'-3')* |
| --- | --- | --- |
| siRNA  Cofilin-1 | Forward | GGGUCAUCAAAGUGUUCAATT |
|  | Reverse | UUGAACACUUUGAUGACCCTT |
| siRNA  Cofilin-2 | Forward | GGAUCAAGCAUGAAUUACATT |
|  | Reverse | UGUAAUUCAUGCUUGAUCCTT |
| siRNA  Cofilin-3 | Forward | CCACCUUUGUCAAGAUGCUTT |
|  | Reverse | AGCAUCUUGACAAAGGUGGTT |

|  | 0h | 1h | 6h | 12h | 24h | 48h |
| --- | --- | --- | --- | --- | --- | --- |
| nsp1 | 31.698 | 30.990 | 30.991 | 30.961 | 30.676 | 30.819 |
|  | 32.853 | 30.767 | 31.033 | 30.584 | 30.721 | 31.264 |
|  | 31.642 | 30.478 | 30.259 | 31.638 | 31.055 | 30.560 |
| nsp5 | 31.811 | 30.192 | 31.041 | 31.548 | 31.137 | 31.105 |
|  | 31.678 | 30.557 | 31.612 | 31.633 | 31.930 | 31.196 |
|  | 31.481 | 31.319 | 31.166 | 30.588 | 31.992 | 31.713 |
| PLP2 | 30.920 | 30.419 | 30.559 | 30.089 | 30.962 | 30.670 |
|  | 30.359 | 30.628 | 30.150 | 30.870 | 30.257 | 31.233 |
|  | 30.069 | 30.994 | 30.997 | 30.466 | 30.794 | 30.650 |
| ORF3 | 31.771 | 32.886 | 32.531 | 32.274 | 32.479 | 32.338 |
|  | 32.007 | 32.882 | 31.752 | 32.097 | 32.562 | 32.661 |
|  | 32.193 | 32.077 | 32.274 | 32.598 | 32.120 | 32.802 |

**Supp table 3 Ct values of PEDV non-structural genes**
